# Developing a population-state decision system for intelligently reprogramming extracellular electron transfer in *Shewanella oneidensis*

**DOI:** 10.1073/pnas.2006534117

**Published:** 2020-08-27

**Authors:** Feng-He Li, Qiang Tang, Yang-Yang Fan, Yang Li, Jie Li, Jing-Hang Wu, Chen-Fei Luo, Hong Sun, Wen-Wei Li, Han-Qing Yu

**Affiliations:** ^a^Chinese Academy of Sciences Key Laboratory of Urban Pollutant Conversion, University of Science and Technology of China, 230026 Hefei, China;; ^b^Department of Environmental Science and Engineering, University of Science and Technology of China, 230026 Hefei, China;; ^c^School of Life Sciences, University of Science and Technology of China, 230026 Hefei, China

**Keywords:** population-state decision, intelligently reprogramming, quorum sensing, extracellular electron transfer (EET), Cr (VI) reduction

## Abstract

The unique extracellular electron transfer (EET) ability has positioned electroactive bacteria (EAB) as a major class of cellular chassis for genetic engineering aimed at favorable environmental, energy, and geoscience applications. However, previous efforts to genetically enhance EET ability have often impaired the basal metabolism and cellular growth due to the competition for the limited cellular resource. Here, we design a quorum sensing-based population-state decision (PSD) system for intelligently reprogramming the EET regulation system, which allows the rebalanced allocation of the cellular resource upon the bacterial growth state. We demonstrate that the electron output from *Shewanella oneidensis* MR-1 could be greatly enhanced by the PSD system via shifting the dominant metabolic flux from initial bacterial growth to subsequent EET enhancement (i.e., after reaching a certain population-state threshold). The strain engineered with this system achieved up to 4.8-fold EET enhancement and exhibited a substantially improved pollutant reduction ability, increasing the reduction efficiencies of methyl orange and hexavalent chromium by 18.8- and 5.5-fold, respectively. Moreover, the PSD system outcompeted the constant expression system in managing EET enhancement, resulting in considerably enhanced electron output and pollutant bioreduction capability. The PSD system provides a powerful tool for intelligently managing extracellular electron transfer and may inspire the development of new-generation smart bioelectrical devices for various applications.

## Significance

The dynamic optimization of the cellular resource allocation of microbes can efficiently enhance their microbial extracellular electron transfer output. Therefore, we develop a quorum sensing-based system to intelligently reprogram the extracellular electron transfer output upon the population state. This system is able to autonomously shift the dominant metabolic flux from the initial microbial growth to the later extracellular electron transfer enhancement. By incorporating the extracellular electron transfer network, this system achieves significant improvements in extracellular electron transfer output and pollutant reduction capability. This work provides a powerful approach to intelligently manage microbial extracellular electron transfer for improved environmental applications and may serve as a feasible and effective tool for the development of smart bioelectrical devices.

The rapid advances in genetic engineering and synthetic biology techniques have given rise to new opportunities for the development of next-generation environmental biotechnology (1–3), which targets hybrid pollutant degradation pathways (4), higher degradation efficiency (5), and an expanded pollutant spectrum (6). However, the full exploitation of the potential of genetically engineered microbes is limited by the metabolic burden caused by the imported foreign gene circuit, the suboptimal allocation of cellular resource, or inconsistency between gene expression and dynamic cellular demand (7, 8). These problems often lead to cellular growth inhibition and metabolic unfitness, impeding the full functioning of engineered cell systems. Such issues are mainly attributed to the insufficient consideration of the complexity and design principles of cellular systems. Differing from an electrical system that functions under a simple 0/1 input control mode (9–11), microbes exhibit high complexity and variability; therefore, elaborated cellular behavior modulation is required to optimize their functionality.

Electroactive bacteria (EAB), which present a unique ability of extracellular electron transfer (EET) (12, 13), show great potential for environmental remediation (14, 15), bioenergy harvesting (16), chemical synthesis (17), and many other applications. To further improve their EET efficiency and application performance, the genetic engineering of these strains has been performed by increasing the intracellular pool of the NADH (nicotinamide adenine dinucleotide hydride) electron carrier (18), promoting the production of the electron shuttle flavin (19), or manipulating cyclic dimeric guanosine monophosphate messenger levels (20). However, these added functions would incur extra consumption of cellular resources, which may competitively impair cellular growth and basal metabolism. Therefore, there is a critical need to maintain a dynamic balance between the cell growth and basal metabolism of EAB and EET enhancement for cellular resource allocation, which has been overlooked previously.

Such a crucial need has motivated us to design a population-state decision (PSD) system to intelligently and dynamically reprogram the EET ability of EAB upon their population state. To this end, we chose *Shewanella oneidensis* MR-1, a model EAB species (21, 22), and harnessed genetic components from the *lux* quorum-sensing (QS) system to construct the PSD system (23). The PSD system applies a cellular resource allocation mechanism that prioritizes initial bacterial growth, with a subsequent shift to EET enhancement. The changes in the EET ability and pollutant bioreduction capacity were evaluated. Furthermore, the performance of the PSD system in managing the electron output was compared with that of the conventional constant expression system.

## Results

### PSD System for Intelligent EET Enhancement.

**Fig. 1.**
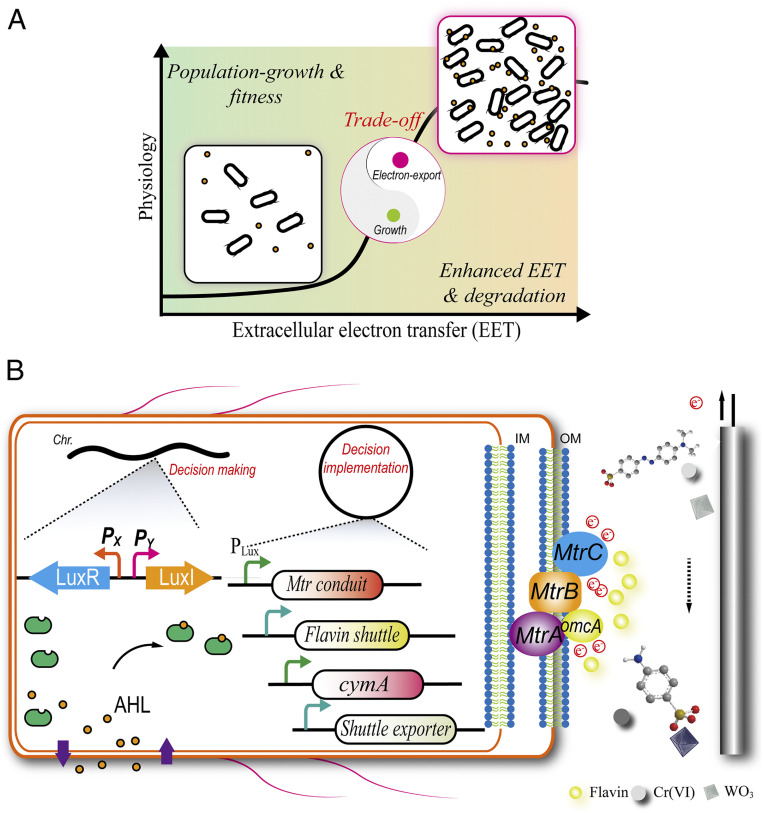
Developing a PSD system for intelligently reprogramming EET. (*A*) A conceptual, system-level design of the PSD system. The PSD system reprograms the cellular resource allocation between the microbial growth and the electron output. (*B*) Diagram of the PSD system for intelligently regulating EET enhancement. Partition of PSD system into two functional parts: the decision-making unit and decision implementation unit.

To maximize the electron output of the EAB population, we designed a PSD system to intelligently reprogram the cellular resource allocation (Fig. 1*A*). With the designed PSD system, more cellular resource was diverted toward cellular growth at this stage. Then, after the bacterial growth of the EAB reached a threshold, the PSD system started to reallocate cellular resource to favor EET enhancement.

The PSD system could be partitioned into two units: the decision-making unit and the decision implementation unit (Fig. 1*B*). The first unit consisted of the reconstructed *luxR* and *luxI*, which encoded the receptor protein and the synthase of *N*-(3-oxohexanoyl)-homoserine lactone (AHL), respectively. Setting at a certain level of the intracellular LuxR, the AHL molecule functioned as the signal to reflect the EAB population status. Upon reaching the AHL concentration threshold, a sufficient amount of the functional LuxR−AHL complex was formed to initiate the P_Lux_-responsive system for decision-making. The second unit was composed of the responsive system and the governed enzymes and pathway genes. Thus, upon reaching a certain population status, the implementation unit would shift more cellular resource to initiate the enhancement of EET output.

### Functions of an Artificial QS System.

**Fig. 2.**
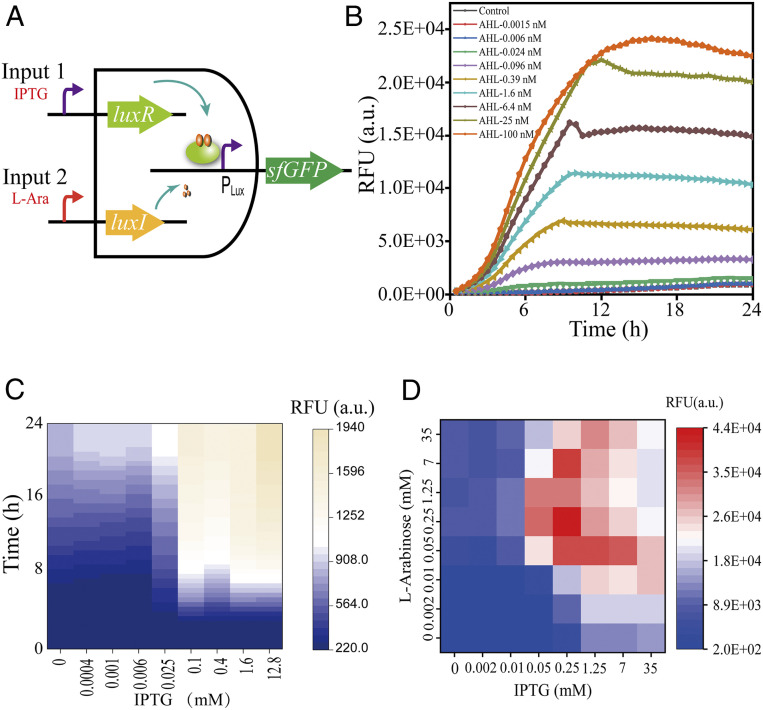
Evaluation of the artificial QS system in *S. oneidensis.* (*A*) Construction of the artificial *luxI-luxR* AND gate. (*B*) System evaluation in response to the exogenous AHL added at different concentrations. (*C*) System sensitivity modulation by tuning the intracellular LuxR receptor level. (*D*) Combinational modulation of the system for various outputs. RFU: relative fluorescence units. Control: wild-type strain with the empty pYYDT plasmid (WT/pYYDT).

The QS system was reconstructed in *S. oneidensis* for evaluation. An artificial “AND” gate was built with both *luxR* and *luxI* under the control of inducible promoters for tunability. The superfolder green fluorescent protein (*sfGFP*) reporter was driven by the responsive P_Lux_ promoter to facilitate the output determination (Fig. 2*A*).

First, we settled the *luxR* at a basal level of expression and supplemented the microbial culture with exogenous AHL. The wild-type strain with the empty plasmid pYYDT was utilized as the control here, and for all of the subsequent experiments as well. As a result, the sfGFP fluorescence increased in response to the increased AHL concentration in the range of 0.024 nM to 100 nM (Fig. 2*B*). Such an AHL-dependent system output suggested the feasibility of tuning the system according to the state of the microbial population as reflected by the AHL level. Next, we fixed the AHL level at a constant level and adjusted the intracellular LuxR level by adding isopropyl β-d-1-thiogalactopyranoside (IPTG) at different concentrations. As shown in Fig. 2*C*, the system was more sensitive to AHL in terms of the system output at a higher LuxR level, whereas it was insensitive at a lower LuxR level, with a threshold at 0.1 mM IPTG induction. Noteworthy is that the system output was almost consistent under 0.1 mM to 12.8 mM IPTG induction, indicating that the system could not be precisely and dynamically tuned by varying the LuxR receptor alone. Therefore, the tunability of the system output in terms of the population state and system sensitivity, by varying *luxI* for AHL synthesis and *luxR* for system sensitivity in combination, was verified, and dynamic and efficient system output was achieved (Fig. 2*D*). Collectively, these results reveal that *lux* QS elements could be used to construct the PSD system for EET enhancement.

### Construction of Decision-Making Unit Implanted Hosts.

**Fig. 3.**
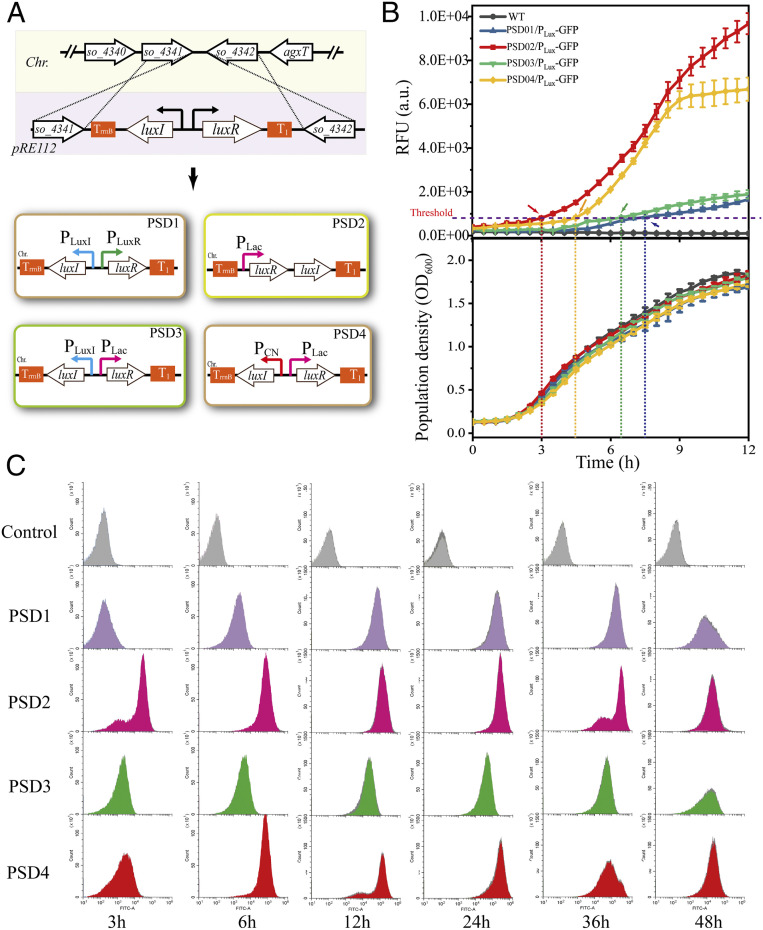
Construction of the PSD system-implanted hosts. (*A*) The procedure for constructing the four various PSD-implanted hosts. (*B*) Intelligent outputs using sfGFP as the reporter (*Upper*) and the biomass accumulation (*Lower*) of the PSD-implanted strains in the initial 0 h to 12 h. The error bars (mean ± SD) are derived from the independent experiments conducted in triplicate for each strain. (*C*) Flow cytometry analysis of the outputs of the PSD system variants at the single-cell level. Flow cytometry experiments were conducted with three independent biological samples for each strain. Control: WT/pYYDT.

To facilitate the utilization of the PSD system, we aimed to construct autonomously regulated decision-making units for the PSD system. To explore the effects of the genetic structure and expression level on the performance of the PSD system, four system architectures were designed. As shown in Fig. 3*A*, in PSD1, the native QS promoters were harnessed for PSD element expression in *S. oneidensis*, and the two cassettes were placed in the opposite direction. In PSD2, the constitutive promoter P_Lac_ was utilized, and a tandem genetic structure was adopted. In PSD3, the *luxI* gene was controlled by its native promoter, while the *luxR* gene was under the control of the P_Lac_ promoter. Lastly, in the design of PSD4, both *luxI* and *luxR* were with constitutive promoters (P_CN_ and P_Lac_, respectively) (24), and they were assembled in the opposite direction. For all four architectures, terminators were placed at both ends of the genetic structures, thereby insulating the genetic structures with the neighboring genetic contexts.

These decision-making units were then implanted into the chromosomal neutral site between *so_4341* and *so_4342* through homologous recombination. Next, these strains were evaluated for the performance of the PSD system (Fig. 3*B* and *SI Appendix*, Fig. S1). PSD2 exhibited the earliest response to the population state, and its system output was the highest. PSD4 initiated the system output after reaching a higher growth level, and its output ranked second. In contrast, both PSD1 and PSD3 initiated the system at much higher population levels and exhibited relatively lower outputs. Furthermore, the flow cytometry analysis of the sfGFP expression levels at the single-cell level shows consistent results with the above population-level data (Fig. 3*C*). During the experimental period, all of the PSD systems exhibited great durability, especially within the initial 36 h. Additionally, the four strains exhibited benign centrality, indicating that few cell-to-cell variations occurred within the microbial population (*SI Appendix*, Fig. S2). These results indicate that the genome-implanted decision-making units could effectively and dynamically initiate the decision implementation unit at various population-state levels and trigger the PSD system output at various levels.

### Intelligent Reprogramming of EET Pathways by the PSD System.

**Fig. 4.**
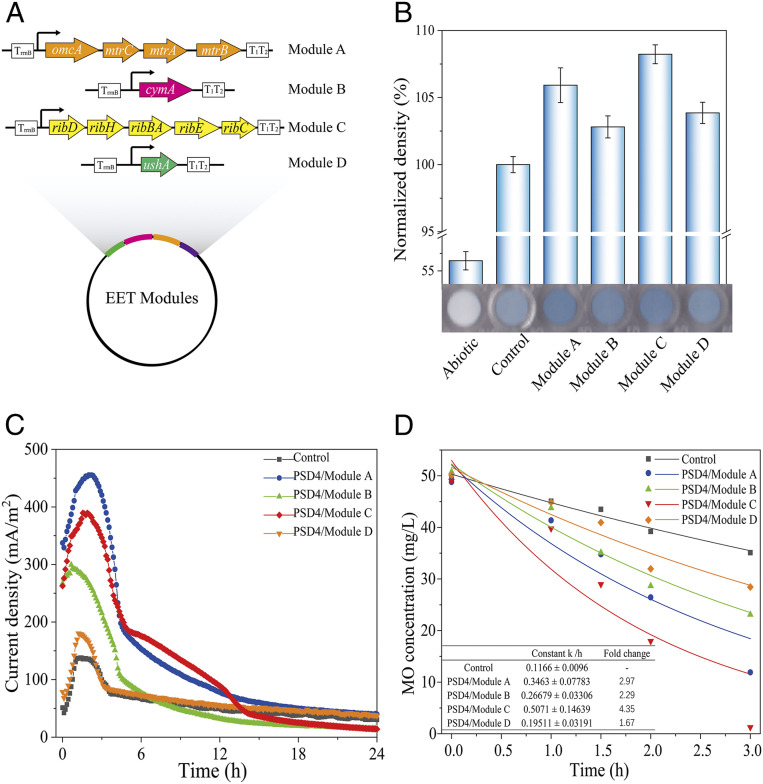
PSD-enabled intelligent reprogramming of the EET pathways. (*A*) Illustration of the PSD-reprogrammed EET pathways. Module A: intelligently reprogrammed OmcA-Mtr pathway. Module B: intelligently reprogrammed CymA. Module C: intelligently reprogrammed flavin synthesis pathway. Module D: intelligently reprogrammed UshA exporter. (*B*) EET evaluation with the WO_3_ test. (*C*) The current outputs of the strains with intelligently reprogrammed pathways and the control evaluated with the MECs. (*D*) Reduction and first-order fitted curves of MO reduction by the intelligently reprogrammed strains and the control within the initial 3 h of the bioreduction experiment. Control: WT/pYYDT. The error bars (mean ± SD) are derived from the independent experiments conducted in triplicate for each strain.

The validity of the PSD system for intelligently controlling the EET pathways was further examined. Here, the strain PSD4 was selected, owing to its greater biomass accumulation before initiating the efficient system output. To intelligently reprogram the EET pathways, four decision implementation modules were designed and constructed to reprogram the Mtr conduit (OmcA-MtrCAB) (25), the tetraheme menaquinol dehydrogenase CymA (26), the electron shuttle flavin synthesis pathway (27), and the periplasmic flavin adenine dinucleotide hydrolase UshA, respectively (Fig. 4*A*) (28). These modules were subsequently subjected to EET ability detection using a WO_3_ probe (29). As anticipated, all four implementation modules exhibited the development of blue color more rapidly than the control, indicating the substantially enhanced EET abilities of these engineered strains (Fig. 4*B*). Then, they were assessed in a microbial electrolysis cell (MEC) system, in which the current densities of all of the groups peaked at ∼2.1 h. The peak current densities of the modules A, B, C, and D were 3.3-, 2.2-, 2.8-, and 1.3-fold higher, respectively, than that of the control (Fig. 4*C*).

Furthermore, we examined the feasibility of using the PSD-reprogrammed strains for the extracellular reduction of methyl orange (MO), a model azo-dye pollutant (30). Consistent with the WO_3_ and MEC results, all of the implementation modules exhibited much higher MO reduction rates than the control (*SI Appendix*, Fig. S3). Their first-order reduction rate constants were 1.7- to ∼4.4-fold over that of the control (Fig. 4*D*). These results demonstrate that the PSD system could be successfully used to intelligently regulate EET pathways to achieve an enhanced electron output and pollutant reduction.

### Systemic Reprogramming of EET Network.

**Fig. 5.**
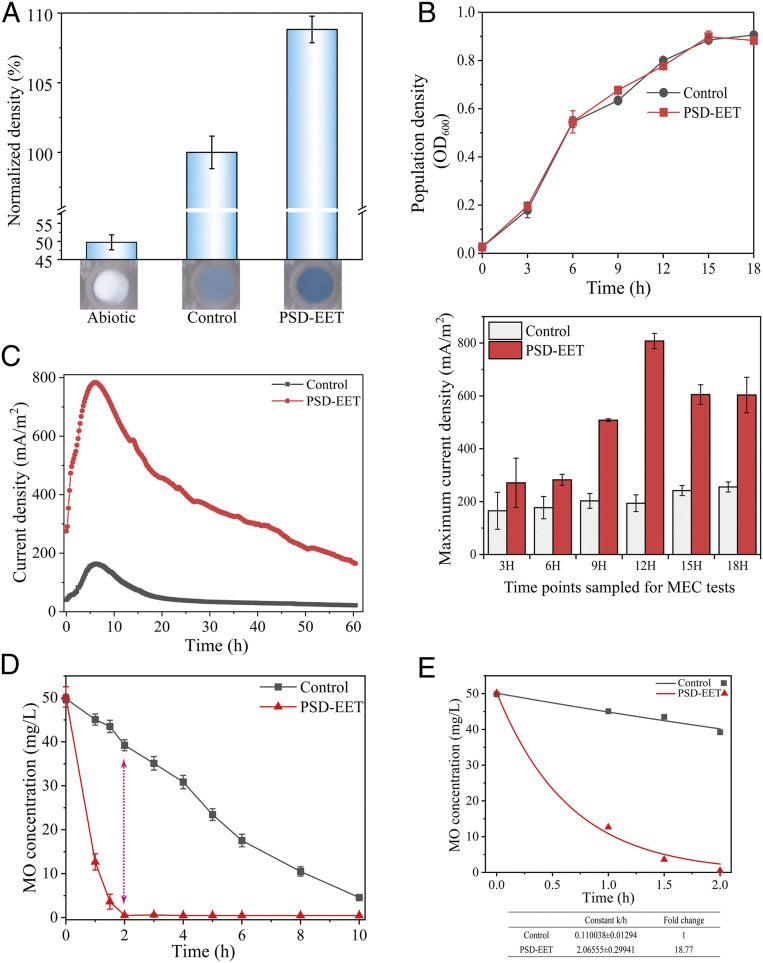
Systemic reprogramming of the EET network by the PSD system. (*A*) EET evaluation with the WO_3_ test for the systemically reprogrammed PSD-EET strain. (*B*) Trade-off between the bacterial growth and EET output. (*Upper*) The bacterial growths of the PSD-EET strain and the control. (*Lower*) The maximum current outputs of the PSD-EET strain and the control, harvested at the indicated time points. (*C*) The current output curves of the PSD-EET and the control strains harvested at 12-h. (*D*) Reduction curves and (*E*) first-order fitted curves of MO reduction by the PSD-EET strain and the control. Control: WT/pYYDT. The error bars (mean ± SD) are derived from the independent experiments conducted in triplicate for each strain.

Owing to the better EET enhancement performances of modules A, B, and C through intelligent regulation, these modules were assembled to systemically reprogram the EET network with the PSD system (designated as strain PSD-EET). The evaluation of the EET ability of this strain with the WO_3_ probe showed a more enhanced electron output over that obtained with the single modules, reflecting a synergistic effect of these intelligently regulated modules (Fig. 5*A*).

Furthermore, we explored the trade-off between the bacterial population state and the EET output. Both the PSD-EET and the control strains were cultivated in mineral medium. At every interval of 3 h, their cells were sampled for cell density examination and also for EET determination with MEC systems. As a result, the PSD-EET strain and the control strain exhibited almost the same growth level (Fig. 5 *B*, *Upper*), suggesting the feasibility of the cellular resource reallocation for bacterial growth by the PSD system. The efficient EET enhancement of the PSD-EET strain over that of the control was initiated from the cell density (optical density at 600 nm [OD_600_]) of 0.65 to ∼0.7, and the greatest enhancement of EET was achieved at OD_600_ of ∼0.8 (Fig. 5 *B*, *Lower*). These results clearly demonstrate the trade-off between the bacterial growth and electron transfer. Specifically, the current output curve of the MEC cultivated with the PSD-EET strain, which was harvested at 12 h, achieved the highest enhancement of EET output over the control. Its maximum current density reached 783 mA/m^2^, which was 4.8-fold higher than that of the control (Fig. 5*C*). The biofilm on the electrodes in the MECs was also examined using scanning electron microscopy (SEM). As shown in *SI Appendix*, Fig. S4, no differences in the biofilm were observed. Moreover, the PSD-EET and the control strains had a similar level of biomass (*SI Appendix*, Fig. S5). These results clearly reveal the feasibility of using the PSD system in managing the cellular resource reallocation to facilitate the maintenance of bacterial physiological fitness. Therefore, uploading the EET genetic network encoded by the PSD system would not cause apparent cell growth defects and physiological unfitness.

Furthermore, the PSD-EET strain also exhibited a higher MO reduction rate (with an 18.8-fold greater degradation rate constant than the control) and completely removed MO within 2 h (Fig. 5 *D* and *E*). Collectively, these results demonstrate that a considerable enhancement in the electron output could be realized by systemically reprogramming the EET network with the PSD system.

### Application of the Reprogrammed Strain for Cr(VI) Reduction.

**Fig. 6.**
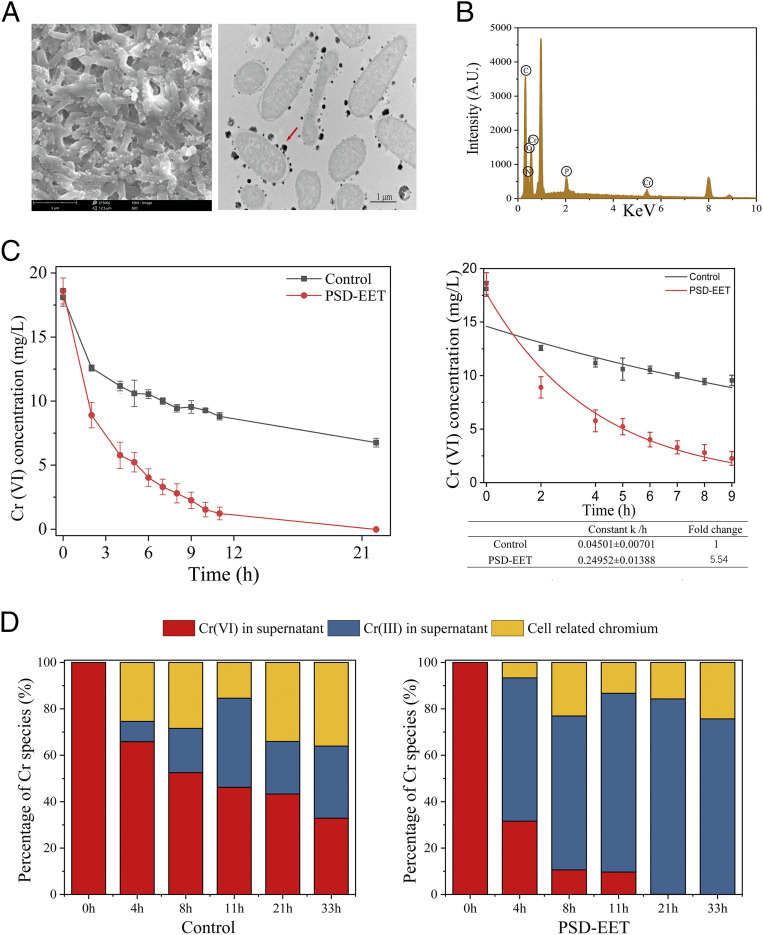
Application of the systemically reprogrammed strain for Cr(VI) reduction. (*A*) SEM image of the chromium precipitate associated with the cell surface (*Left*) and TEM image of the ultrathin sections produced with an ultramicrotome after Cr(VI) exposure to strain PSD-EET (*Right*). (*B*) EDS analysis of the formed chromium precipitate. (*C*) Reduction curves (*Left*) and first-order fitted curves (*Right*) of Cr(VI) reduction by the PSD-EET strain and the control. (*D*) Distribution analysis of Cr species after Cr(VI) reduction by the PSD-EET strain and the control. Control: WT/pYYDT. The error bars (mean ± SD) are derived from the independent experiments conducted in triplicate for each strain.

The performance of the PSD-EET strain for enhanced pollutant reduction was validated by using the heavy metal Cr(VI) as the model (31). The SEM and transmission electron microscopy (TEM) images reveal the formation of granulated precipitates on the cellular surface (Fig. 6*A* and *SI Appendix*, Fig. S6) and in the surrounding environment (*SI Appendix*, Fig. S7) after the reaction. The energy-dispersive X-ray spectroscopy (EDS) analysis confirms the presence of chromium in the precipitates (Fig. 6*B*). Moreover, the X-ray photoelectron spectroscopy (XPS) analysis shows the strong peaks with binding energies of 577.70 and 587.65 eV (*SI Appendix*, Fig. S8), attributed to the 2p_3/2_ and 2p_1/2_ orbitals of the Cr(III) shell, respectively (32). These results demonstrate the efficient reduction of Cr(VI) by the PSD-EET strain.

The PSD-EET strain exhibited a stronger Cr(VI) reduction capacity than the control. The Cr(VI) reduction rate constant (*k*) value was 5.5-fold higher than that of the control (Fig. 6*C*). Accordingly, more Cr(III) and less Cr(VI) were detected in the supernatant of the PSD-EET group (Fig. 6*D*). Altogether, these results demonstrate that the intelligent reprogramming of the EET network with the PSD system was an effective strategy for enhancing the EET and pollutant bioreduction abilities of *S. oneidensis* MR-1.

### Comparison of the PSD System with a Constant Expression System in Managing Electron Output.

**Fig. 7.**
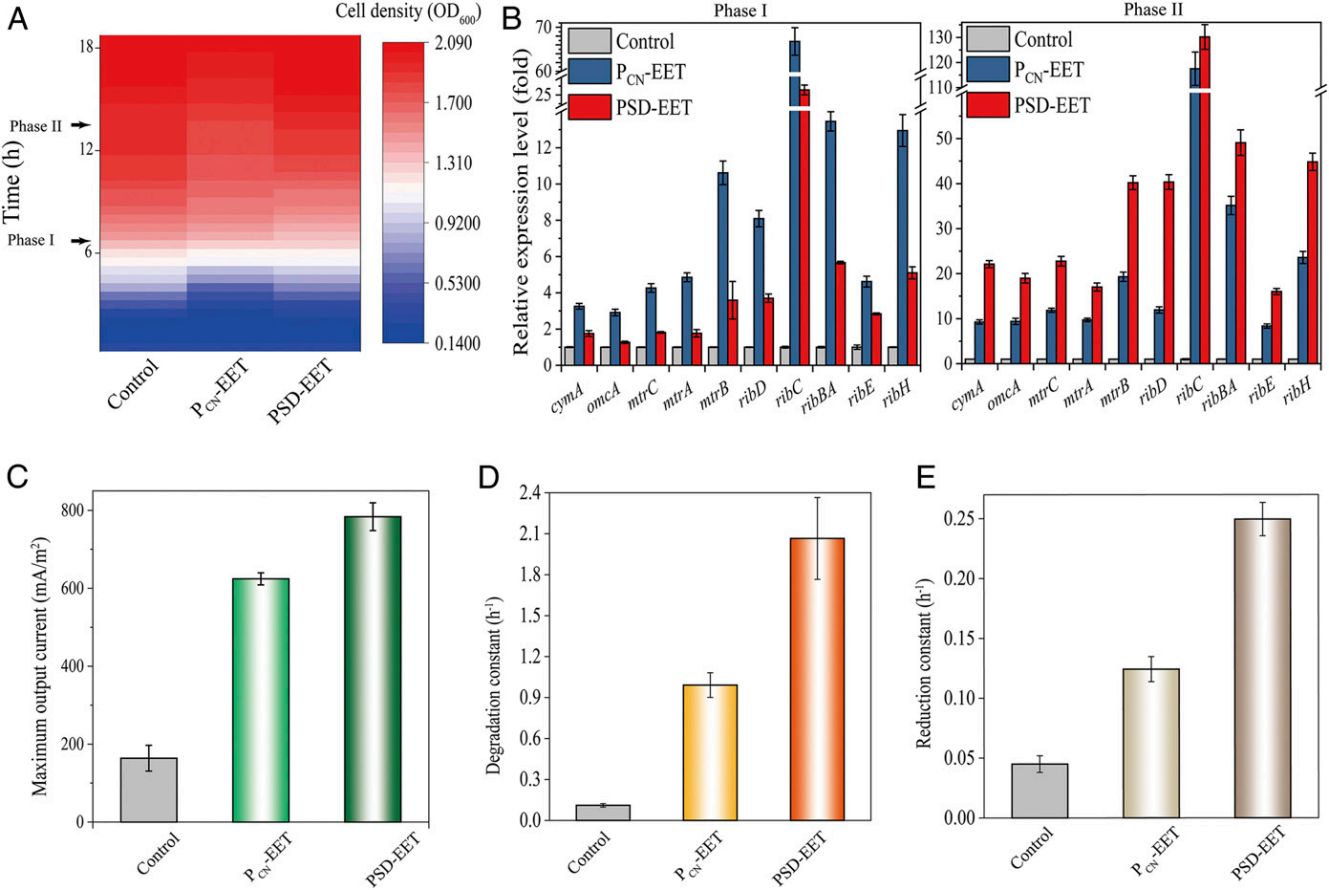
Comparison of the PSD system with the constant expression system in EET management. (*A*) Comparison of the growths of the PSD-EET strain, the P_CN_-EET strain, and the control (in 2×YT medium). (*B*) Transcription level determination of EET genes reprogrammed by the PSD system and the constant P_CN_ expression system. (*Left*) Sampled at phase I. (*Right*) Sampled at phase II. (*C*) The maximum output currents, (*D*) MO degradation rates and (*E*) Cr(VI) reduction rates of the two strains with EET reprogrammed by the PSD system and P_CN_ based constant system, respectively. Control: WT/pYYDT. The error bars (mean ± SD) are derived from the independent experiments conducted in triplicate for each strain.

The PSD system was compared with the conventional gene expression system for managing electron output. The constant expression system of the strong constitutive promoter P_CN_ was harnessed to drive the corresponding EET network (designated as strain P_CN_-EET) (24), as both systems required no inducer. As a result, the P_CN_-EET strain showed a slight defect in biomass accumulation compared to the PSD-EET strain and the control (Fig. 7*A*). This result should be attributed to the metabolic burden caused by the constant expression of EET network genes. Next, the gene expression patterns of the two systems were examined. As expected, substantially increased expression of EET network genes was observed in both engineered strains compared to the control (Fig. 7*B*). Noteworthy, the PSD system exhibited a dynamic regulation pattern. Although the expression up-regulation associated with the PSD system was lower than that under P_CN_ initially (phase I), the PSD system exceeded the P_CN_ system in the late phase (phase II). This result demonstrates the intelligent characteristics and advantages of the PSD system over the conventional constant expression systems.

Moreover, the PSD-EET strain exhibited a much more efficient electron output and pollutant degradation than the P_CN_-EET, which also exhibited improved abilities over the control (*SI Appendix*, Figs. S9–S11). Specifically, the maximum output current of the MEC with P_CN_-EET was only 79.6% of that with PSD-EET (Fig. 7*C*). The biofilms formed on the electrodes were examined (*SI Appendix*, Fig. S12). The PSD-EET strain and the control exhibited almost the same thickness of biofilms, whereas the P_CN_-EET strain formed thinner biofilm in comparison. This result is in agreement with the measured biomass shown in Fig. 7*A*, further confirming the superiority of the PSD system, in intelligently regulating cellular resource reallocation, over the conventional system for biomass accumulation. Furthermore, when the strains were applied in reducing MO and Cr(VI), the reduction rate constant values achieved by the P_CN_-EET strain were only 48.0% and 49.8% of those for the PSD-EET inoculated system (Fig. 7 *D* and *E*). Therefore, the PSD system was more efficient in EET enhancement and pollutant reduction than the constant expression system.

## Discussion

The PSD system is developed to intelligently regulate cellular resource allocation using the parts from the bacterial *lux* QS system in this work. The above results demonstrate that such a PSD system can be used to dynamically and autonomously tune the metabolic flux upon the bacterial population state. This system is applied to intelligently reprogram the EET network of EAB and achieve substantial enhancements in electron output and pollutant reduction capability. In comparison with the conventionally constant system, the PSD system demonstrates its superiority in dynamic and efficient gene regulation and maintenance of the bacterial population physiological fitness.

One of the most attractive features of the PSD system is its ability to optimize the cellular resource allocation in an intelligent fashion. Specifically, the microbial metabolic flux is reallocated from favoring cellular growth in the initial phase to prioritizing EET enhancement in the later phase (Figs. 4*C* and 5 *B* and *C*). In this way, the microbial metabolic flux always targets the limiting factors for electron output. Owing to its intelligent regulation, the metabolic flux is focusing on the bacterial growth in the early phase, avoiding the metabolic burden and unfitness caused due to the constant and strong expressions, which occurs in the conventionally constant system (Fig. 7*A* and *SI Appendix*, Fig. S12).

Varying the genetic structures and modulating the gene expressions of *luxI* for AHL synthesis and *luxR* for system sensitivity is an effective approach to tune the system output. We designed a total of four different architectures for the PSD system (Fig. 3*A*), and their outputs covered various strengths (Fig. 3 *B* and *C*). Among them, the designs of PSD2 and PSD4 initiated the PSD system earlier and achieved more efficient outputs, and the designs of PSD1 and PSD3 initiated the system at higher population biomass, and the outputs would be lower. The outputs of various strengths allow for the selection of the appropriate PSD system variant upon specific needs. For example, when expressing enzymes or pathways, which could be toxic or induce a heavy metabolic burden on the host, the PSD1 and PSD3 would be more suitable.

A further precise tuning of the PSD system requires genetic regulatory elements of different strengths. Although molecular tools have been advanced recently in *S. oneidensis* (33, 34), the well-refined expression elements, for example, promoters and ribosome-binding sequence, are still limited in *S. oneidensis*, which restricts the construction of a set of PSD variants covering wide-ranging output strengths. Therefore, it is essential to explore and construct a kit of a plentiful number of regulatory elements in *S. oneidensis* and other EAB species, and utilize them in combination to modulate and customize the PSD system outputs for different purposes.

The gene expression control system is the basis of microbial genetic engineering, but this factor is usually neglected in environmental biotechnologies for engineered strains (35, 36). The commonly adopted inducible expression system requires the dose of inducers to be modulated with temporal precision and usually functions at high dosages, which not only increases costs but also adds to system complexity. Meanwhile, the constant expression system often suffers from a low expression strength (37). Our engineered PSD system requires no inducers and can achieve efficient gene expression in a dynamic manner, thereby rendering it a more feasible and useful tool for environmental applications. QS systems have been adopted for recombinant protein production (38), cell density control (39), and production of human goods (40). Here, we demonstrate the feasibility of harnessing the QS-based PSD system for EET reprogramming, which might provide a powerful approach for constructing sophisticated bioelectric systems.

In summary, we have developed a PSD system to intelligently reprogram the electron output. This system allows the dynamic allocation of the cellular resource to favor initial microbial growth and subsequent EET enhancement. The EET-reprogrammed strain exhibits substantially enhanced electron output and pollutant degradation capabilities and, thus, presents great potential for environmental biotechnologies. The engineered PSD-based intelligent reprogramming approach could be further expanded to other microorganisms and might inspire the development of smart bioelectrical devices for environmental and other applications.

## Experimental Methods

### Bacterial Strains and Cultivation Conditions.

All of the bacterial strains and plasmids used in this work are listed in *SI Appendix*, Table S1. *Escherichia coli* strains were cultured in 2×YT medium (16 g/L tryptone, 10 g/L yeast extraction, and 5 g/L NaCl) at 37 °C. *S. oneidensis* strains were cultured in 2×YT medium or mineral medium at 30 °C. The preparation of mineral medium is described in detail in *SI Appendix*. When necessary, antibiotics at appropriate concentrations were added: for *E. coli*, 50 μg/mL kanamycin, 34 μg/mL chloramphenicol; for *S. oneidensis,* 50 μg/mL kanamycin, 20 μg/mL chloramphenicol. The inducers of IPTG and l-arabinose at the concentrations as indicated were added. The 2,6-diaminophenedioic acid (DAP) at a final concentration of 50 μg/mL was dosed for cultivating *E. coli* WM3064, in which RP4 Tra function is integrated into the genome and is auxotrophic for DAP (41).

### Genetic Manipulation, Plasmid Construction and Conjugation, and Host Construction.

The genomic DNA isolation, plasmid DNA extraction, RNA extraction, and qRT-PCR were performed following the standard protocols or the instructions of the commercial manufacturers, which are detailed in *SI Appendix*, *Supplementary Methods*.

The Gibson mix was prepared and used for plasmid construction (42). *E. coli* neb10β was used for routine plasmid construction and maintenance of plasmids. The mix was introduced into *E. coli* neb10β through electroporation. Both restriction digestions and Sanger sequencing were conducted to validate the resultant plasmids. Plasmids were introduced into *S. oneidensis* cells via conjugation with *E. coli* WM3064 as the donor cells. The preparations of *E. coli* electrocompetent cells, electroporation, and conjugation are detailed in *SI Appendix*, *Supplementary Methods*.

All primers used in this work are listed in *SI Appendix*, Table S2. The details about the construction of the PSD system-encoding plasmids and decision-making unit implanted strains are described in *SI Appendix*, *Supplementary Methods*.

### Evaluation of the PSD System.

To evaluate the artificial QS system and the PSD system, the *sfGFP* gene was harnessed as the reporter. At the population level, the sfGFP output was measured by the microplate reader (Synergy H1, BioTek Co.) (excitation at 485 nm; emission at 528 nm). The single-cell fluorescence analysis was performed by the flow cytometer (CytoFLEX, Beckman Coulter, Inc.). The single-cell fluorescence analysis (excitation at 488 nm, emission at 525 nm) was performed by the flow cytometer (CytoFLEX, Beckman Coulter Inc.). Approximately 50,000 cells per sample were detected and analyzed by CytExpert (v2.3) software (Beckman Coulter, Inc.).

### Analysis of EET Output.

*S. oneidensis* strains were harvested, mixed with the WO_3_ probe, and loaded on the 96-well plate in an anaerobic chamber (DG250, Don Whitley Scientific Co.) (29). The color development of the mixture was monitored with a scanner to quantitatively determine the EET abilities. The density of color development in each well was analyzed using the Image-Pro Plus software (Media Cybernetics Co.). The abiotic control group with only the pure culture medium was applied to eliminate interferences of the abiotic factors.

The MEC experiments were conducted to further validate the EET ability of the strains. The overnight bacterial cultures were inoculated at 1.0% (vol/vol) into mineral medium, and the corresponding antibiotic was supplemented. They were cultured aerobically at 30 °C and 200 rpm for the indicated periods of time. They were transferred into the three-electrode MECs, and 20 mM lactate was replenished to supply sufficient electron donor. Then, the cultures were sparged with a N_2_/CO_2_ (80:20) gas mixture to achieve an anaerobic environment. After that, the MECs were connected with an electrochemical workstation (CHI1030B, Chenhua Instrument Co.), which served as the potentiostat. The Ag/AgCl reference electrodes were used in saturated potassium chloride (KCl) electrolyte. The carbon felt anode served as the only terminal electron acceptor, and a potential of 0.2 V (vs. Ag/AgCl) was applied to the electrodes (2 × 2 cm^2^).

The cell density of the cultures was determined by the spectrophotometry method via measuring OD_600_ in either 2×YT medium or mineral medium. The biomass of the biofilm on the MEC electrodes was analyzed by measuring the protein concentrations. Carbon felts of the same area (1 cm × 1 cm) were cut from the MEC and gently rinsed with phosphate-buffered saline (PBS). NaOH solution (1 mol/L) was used to lyse the attached cells at 95 °C for 10 min. Then, these supernatants were determined with the Micro BCA Protein Assay Kit (Sangon Biotech Co.) according to the manufacturer’s instructions. The biofilm on the electrodes of MECs was observed by SEM (Phenom ProX Desktop SEM, Phenom Scientific Co.). A piece of carbon felt, from the MEC electrodes, was rinsed with PBS and soaked in 2.5% (wt/vol) glutaraldehyde solution over 12 h. After that, the carbon felts were dehydrated with gradient ethanol (25%, 50%, 75%, 90%, and 100%, each gradient soaking for 20 min). Finally, the surface of the gold-sputtered carbon felts was observed by the SEM. Moreover, the biofilm on the electrodes in the MECs was visualized by an inverted confocal laser scanning microscopy (FV1000, Olympus Inc.). A piece of carbon felt, from the MEC electrodes, was rinsed with PBS and soaked in the solution of the green biofilm cell stain (FM 1-43, Invitrogen Co.) at 30 °C for 30 min. After that, the sample was gently rinsed with sterilized water for observation via a 10× or 20× objective.

### Bioreduction Capacity Tests.

The overnight cultures of *S. oneidensis* strains were harvested and injected into the mineral medium, which was prepared to create an anaerobic environment by sparging with a N_2_/CO_2_ (80:20) gas mixture. Then, the filter-sterilized stock solution of MO was added at a final concentration of 50 mg/L. The samples were collected in an anaerobic chamber at given time intervals. The centrifugations were then performed, and the supernatants were used for measurement using the spectrophotometric method at 465 nm (43).

For the Cr(VI) bioreduction tests, the Cr(VI) stock solution was prepared with K_2_Cr_2_O_4_ (chromium at the concentration of 2 g/L) and sterilized with filtration. The fresh *S. oneidensis* strains were supplemented with Cr(VI) to a final concentration of 20 mg/L. Samples were then taken at given time intervals. The Cr(VI) concentration of supernatants was determined using the 1,5-diphenylcarbazide (DPC) method (44). The colorimetric reagent DPC was added to form a violet complex with Cr(VI), which was then analyzed at 540 nm. The total Cr species of the supernatants was analyzed through oxidization of all Cr species into Cr(VI) with KMnO_4_ at 100 °C, and then the DPC method was applied. After the Cr bioreduction experiment, the cultures were harvested via centrifugation, and the samples were resuspended with glutaraldehyde to a final concentration of 2.5% (wt/vol). Then, they were subjected to SEM/EDS analysis (JSM-6700F, JEOL Inc.). Furthermore, the samples of ultrathin sections were prepared by an ultramicrotome for TEM imaging (JEM-2011, JEOL Inc.). High-resolution XPS (ESCALAB 250, Thermo-VG Inc.) was also used to analyze the valence of Cr.

## Supplementary Material

Supplementary File

## Data Availability

All data are included in the text and *SI Appendix*. The bacterial strains and plasmids are available on request.
