# Supplementary material for: Developing a population-state decision system for intelligently reprogramming extracellular electron transfer in Shewanella oneidensis
Source: Proc Natl Acad Sci U S A. 2020 Aug 27;117(37):23001–10. doi: 10.1073/pnas.2006534117 (PMC7502708; doi:10.1073/pnas.2006534117)
Supplement: Supplementary File [file pnas.2006534117.sapp.pdf]

Supplementary Information

Developing a population-state decision system for intelligently reprogramming extracellular electron transfer in *Shewanella oneidensis*

Feng-He Li[†], Qiang Tang[†,*], Yang-Yang Fan[‡], Yang Li[‡], Jie Li[†], Jing-Hang Wu[†], Chen-Fei Luo[‡], Hong Sun[‡], Wen-Wei Li[‡], Han-Qing Yu[†,*]

[†]CAS Key Laboratory of Urban Pollutant Conversion, Department of Environmental Science and Engineering, [‡]School of Life Sciences, University of Science and Technology of China, Hefei, 230026, China

This supplementary information (SI) includes:

- Supplementary Figures
- Supplementary Tables
- Supplementary Methods
- Supplementary References

www.pnas.org/cgi/doi/10.1073/pnas.2006534117

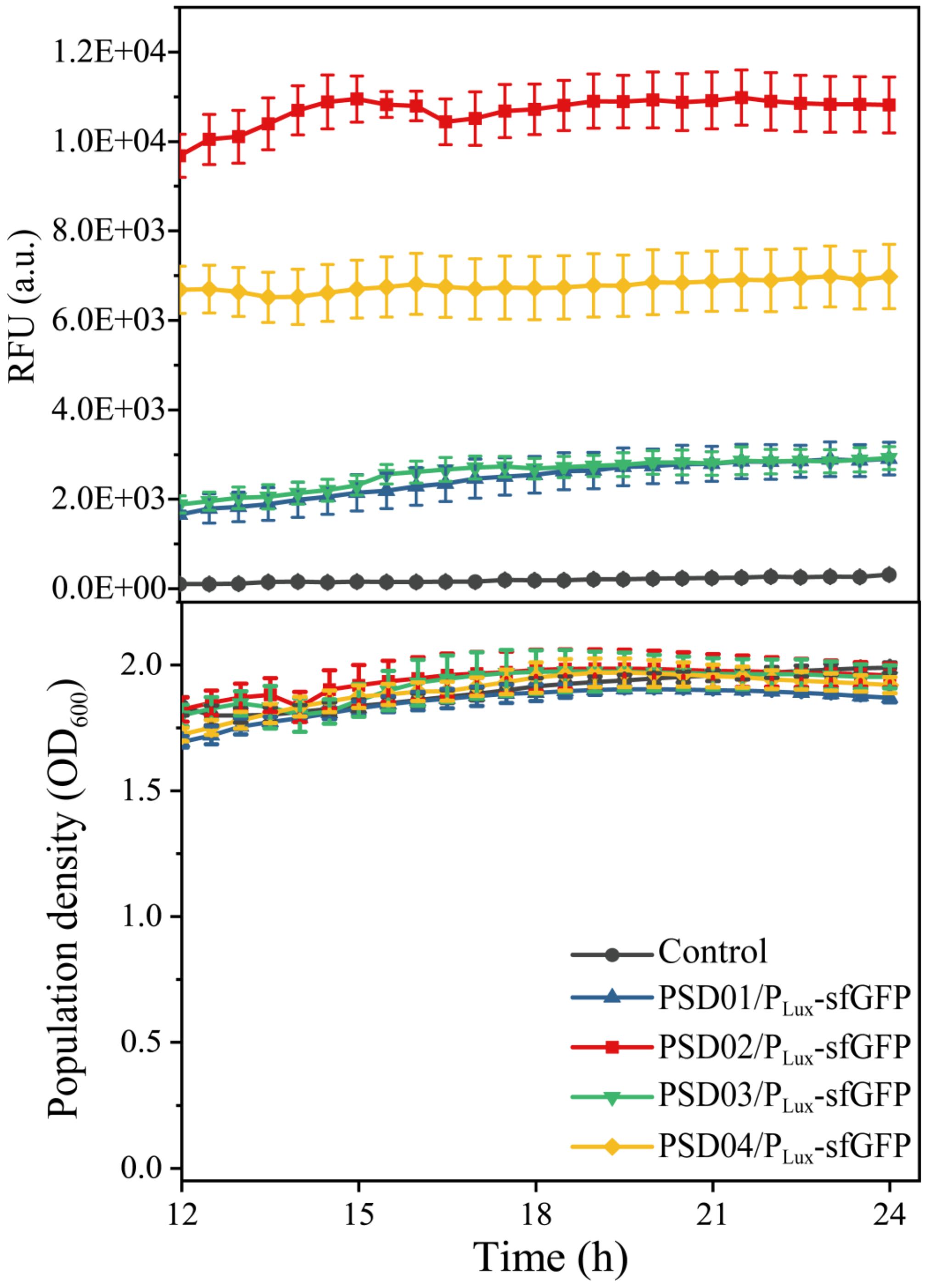

Fig. S1. Intelligent outputs using sfGFP as the reporter (upper panel) and bacterial growth (lower panel) of the PSD-implanted strains during the period of 12-24 h. (n=3 independent replicates). Control: WT/pYYDT.

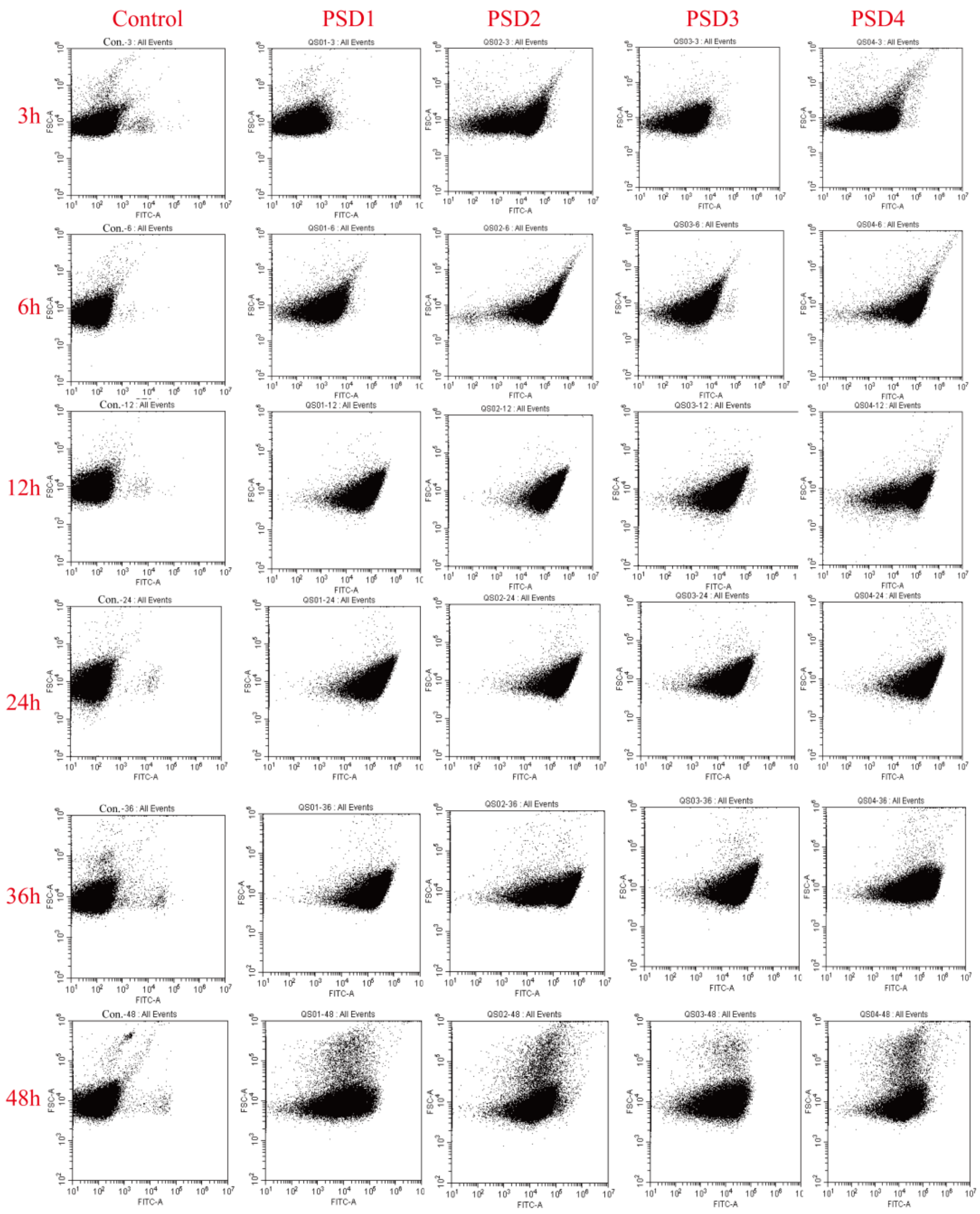

Fig. S2. Evaluation of the cell-to-cell variations within the bacterial population. Control: WT/pYYDT.

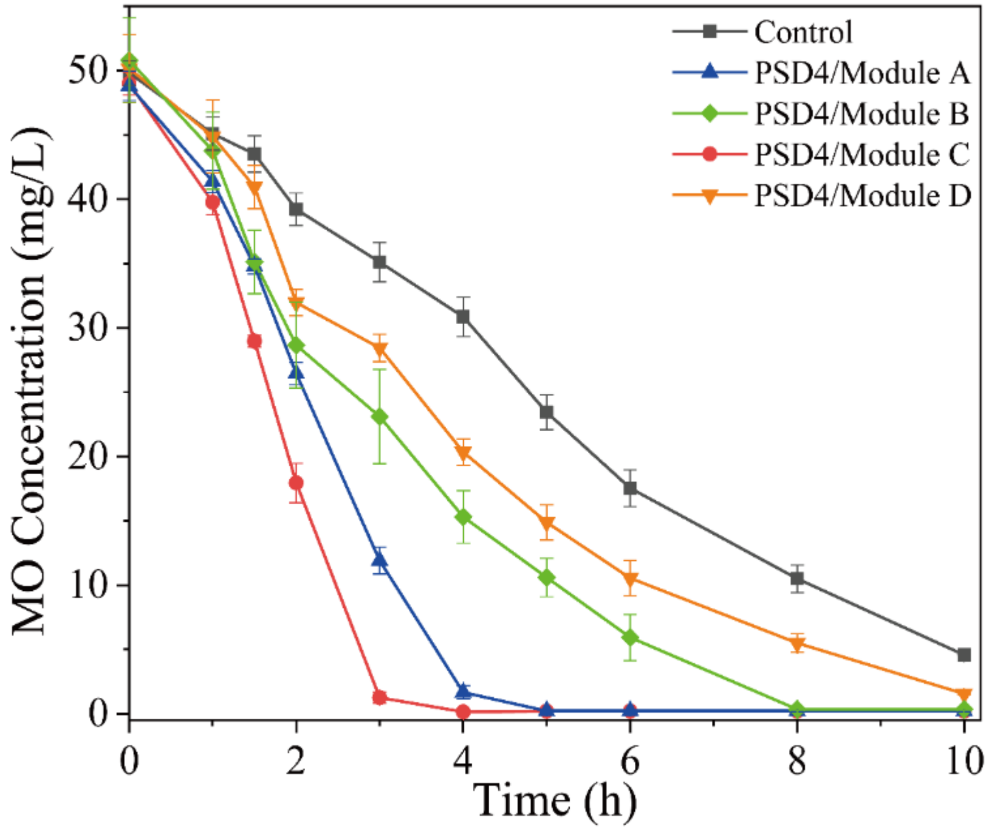

Fig. S3. Reduction curves of MO by the intelligently reprogrammed strains and the control. The error bars (mean ± SD) are derived from the independent experiments conducted in triplicate for each strain. Control: WT/pYYDT.

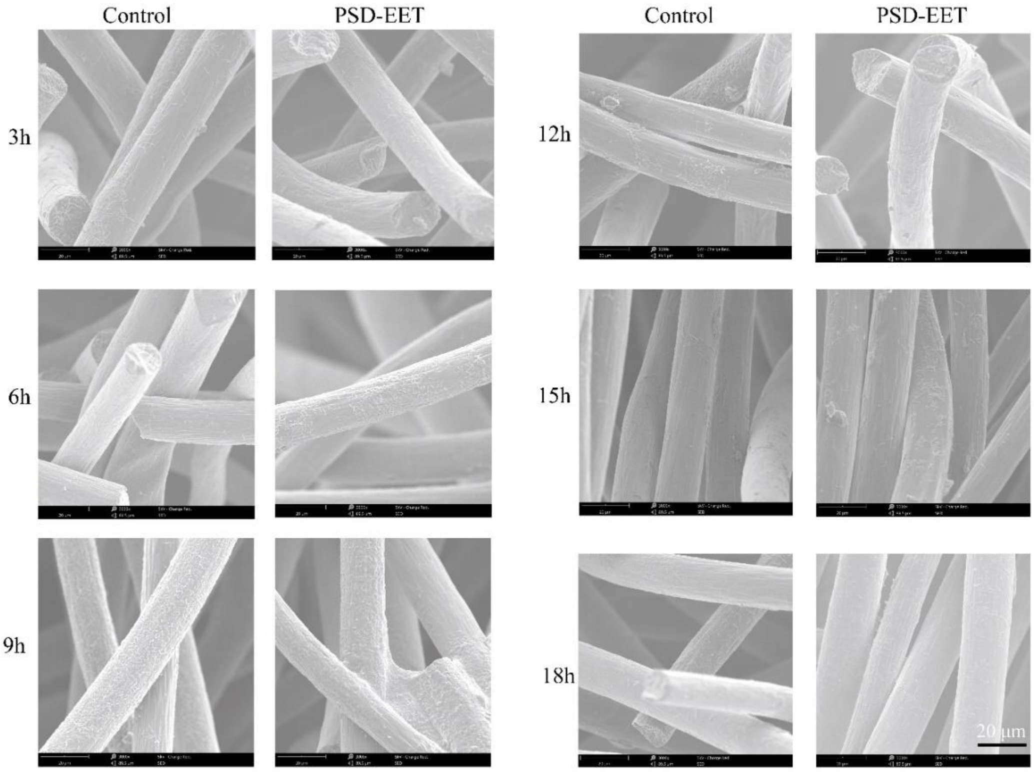

Fig. S4. Monolayer of the biofilm formed by the PSD-EET strain and the control (WT/pYYDT) on the carbon felts.

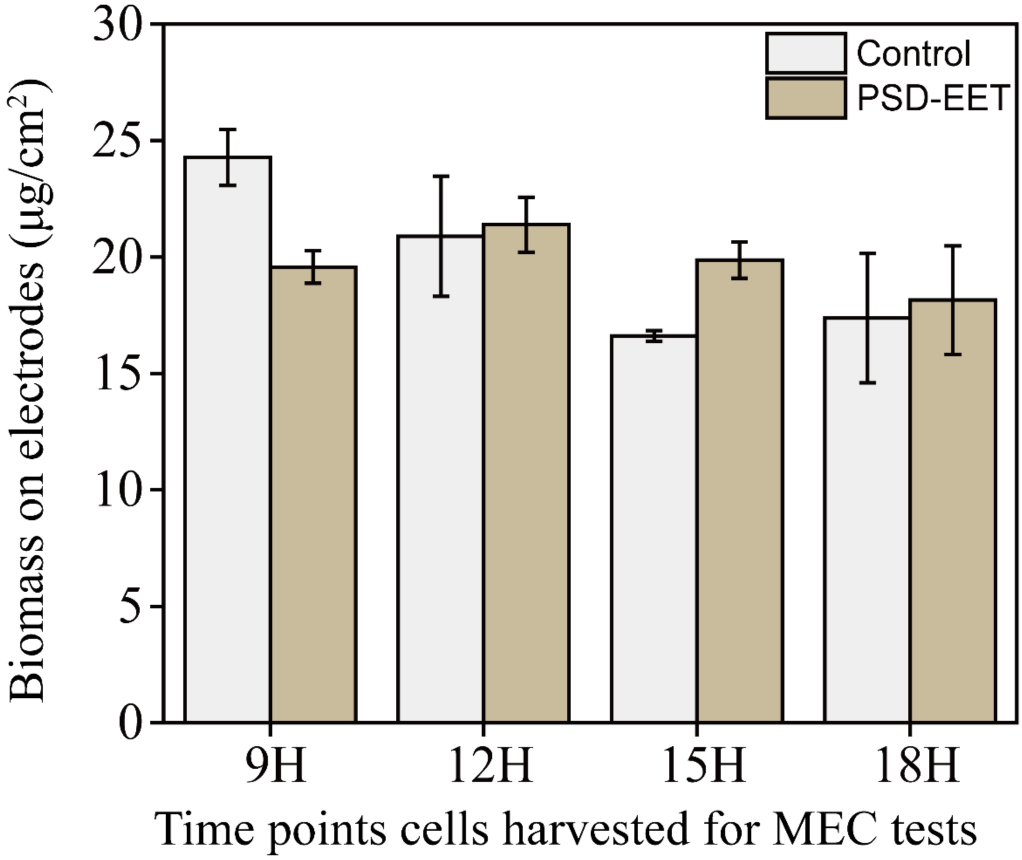

Fig. S5. Determination of the biomass on the electrodes. The error bars (mean ± SD) are derived from the independent experiments conducted in triplicate for each strain. Control: WT/pYYDT.

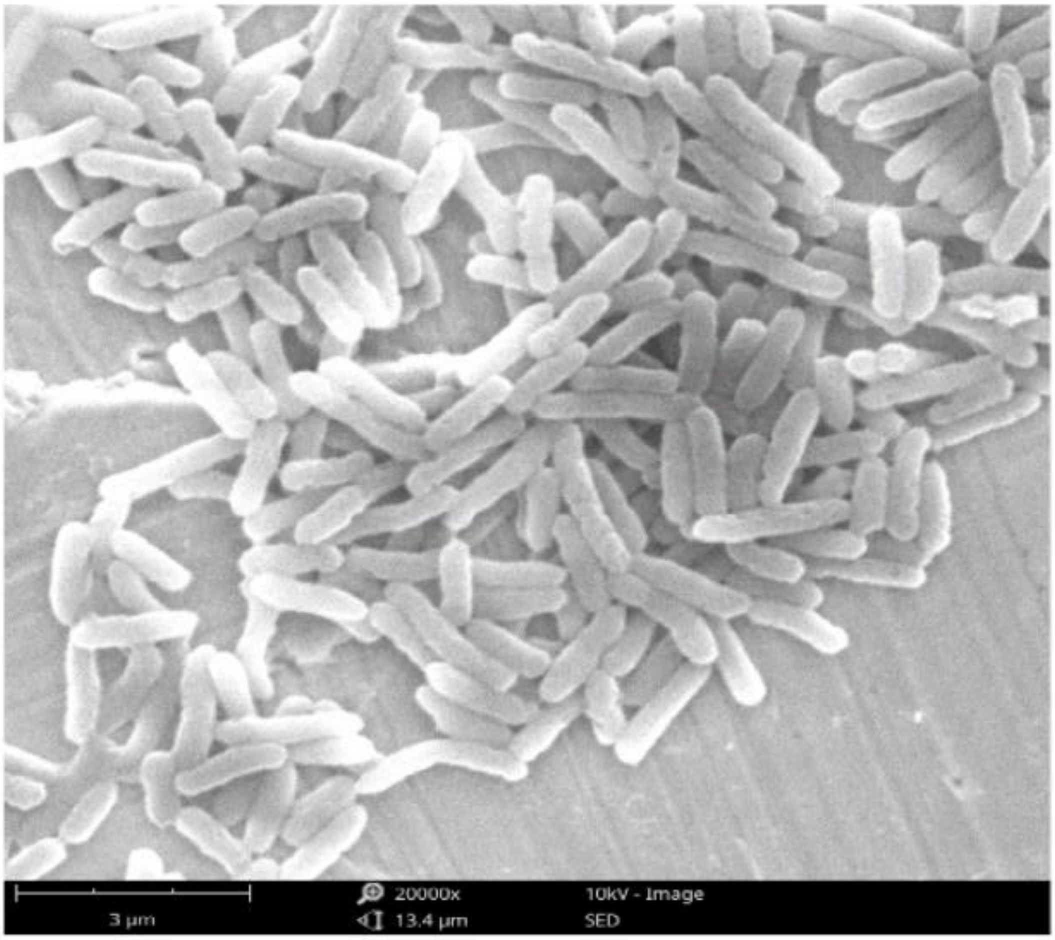

Fig. S6. Smooth cell surface of the strain PSD-EET before exposure to Cr(VI) with SEM imaging.

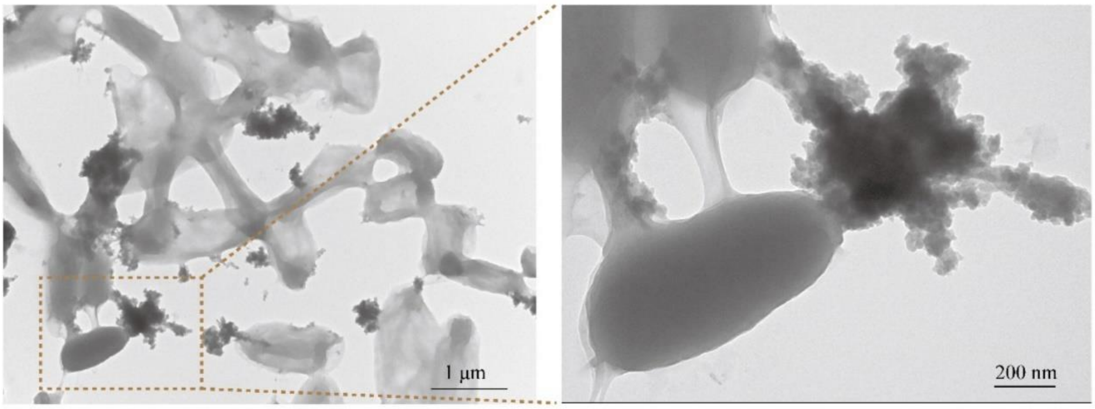

Fig. S7. TEM images of the chromium precipitate on the cell surface and between cells.

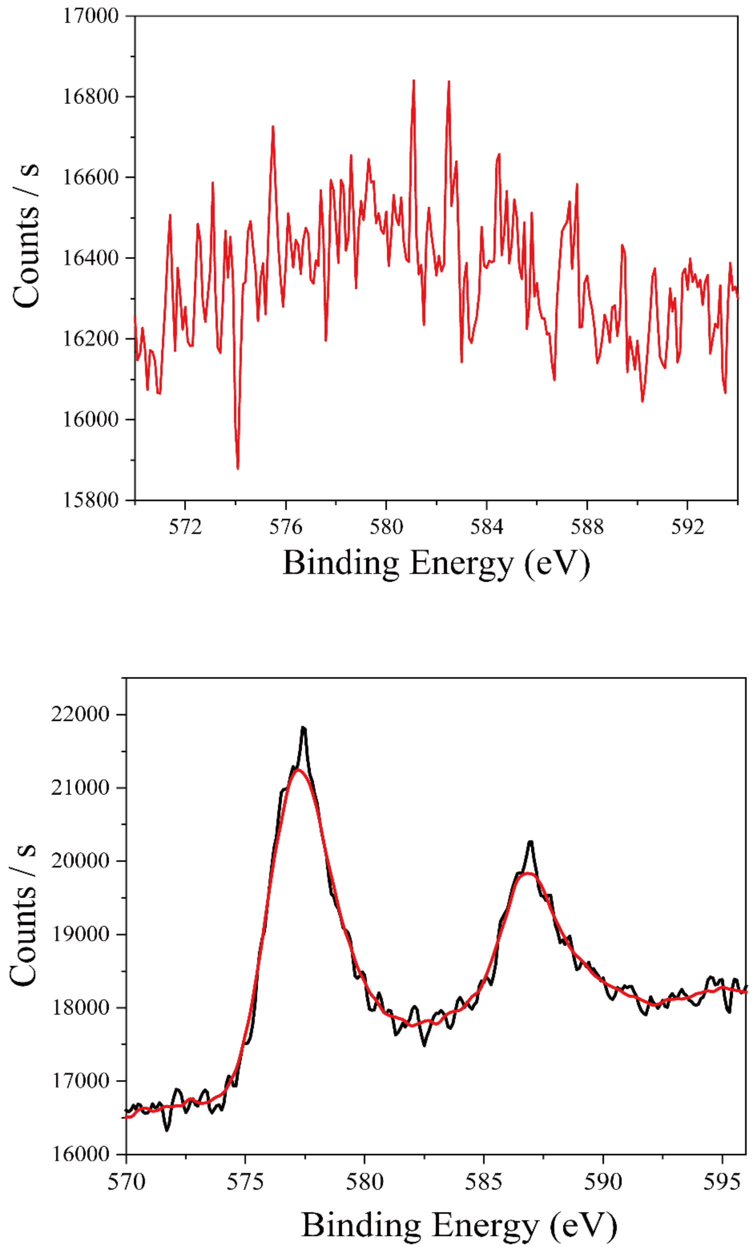

Fig. S8. XPS analysis of the strain PSD-EET before and after exposure to Cr(VI).

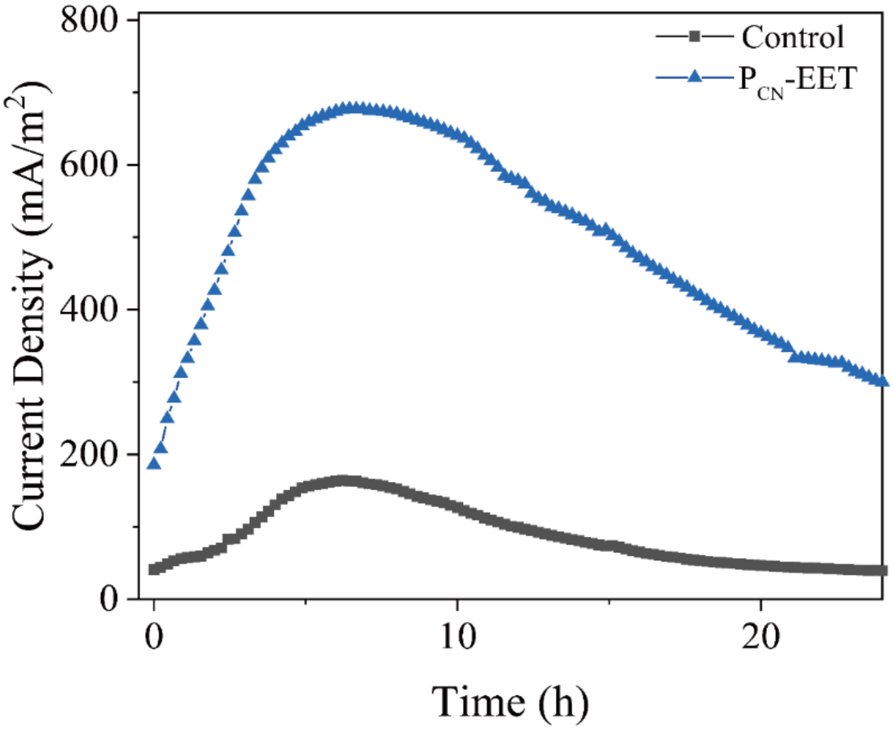

Fig. S9. MEC output of the strain P_{CN}-EET. Control: WT/pYYDT.

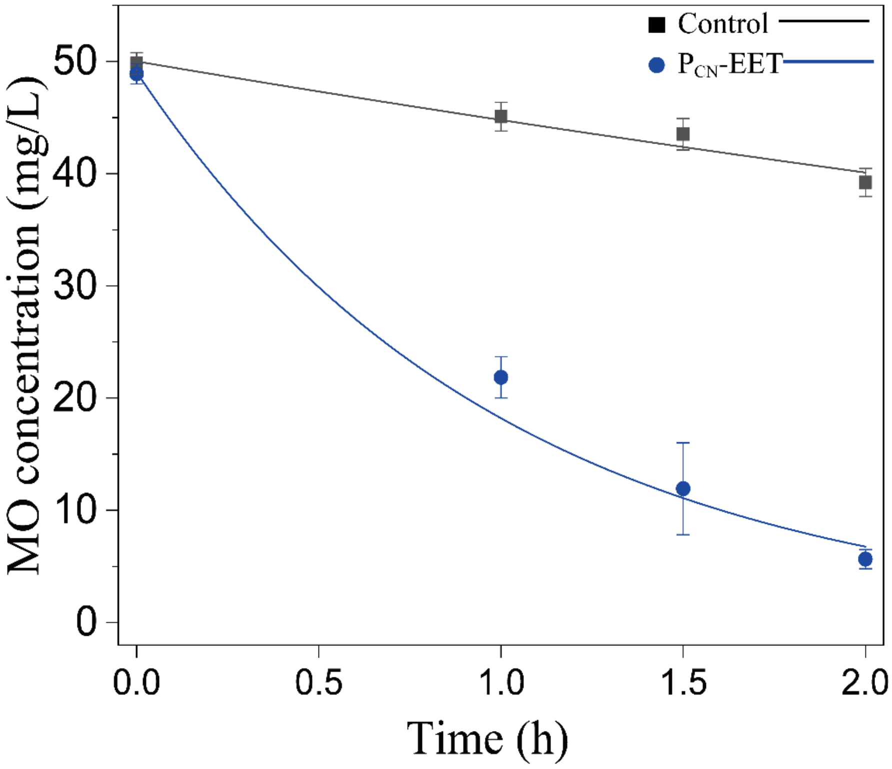

Fig. S10. Bioreduction of MO by the strain P_{CN}-EET. Control: WT/pYYDT.

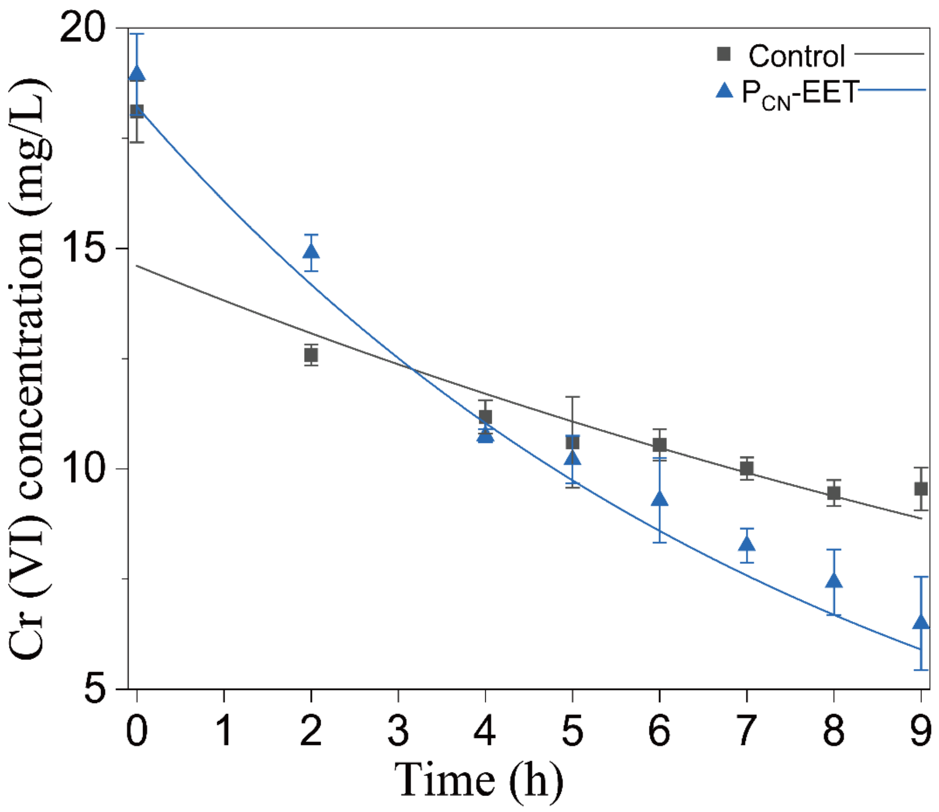

Fig. S11. Bioreduction of Cr(VI) by the strain P_{CN}-EET. Control: WT/pYYDT.

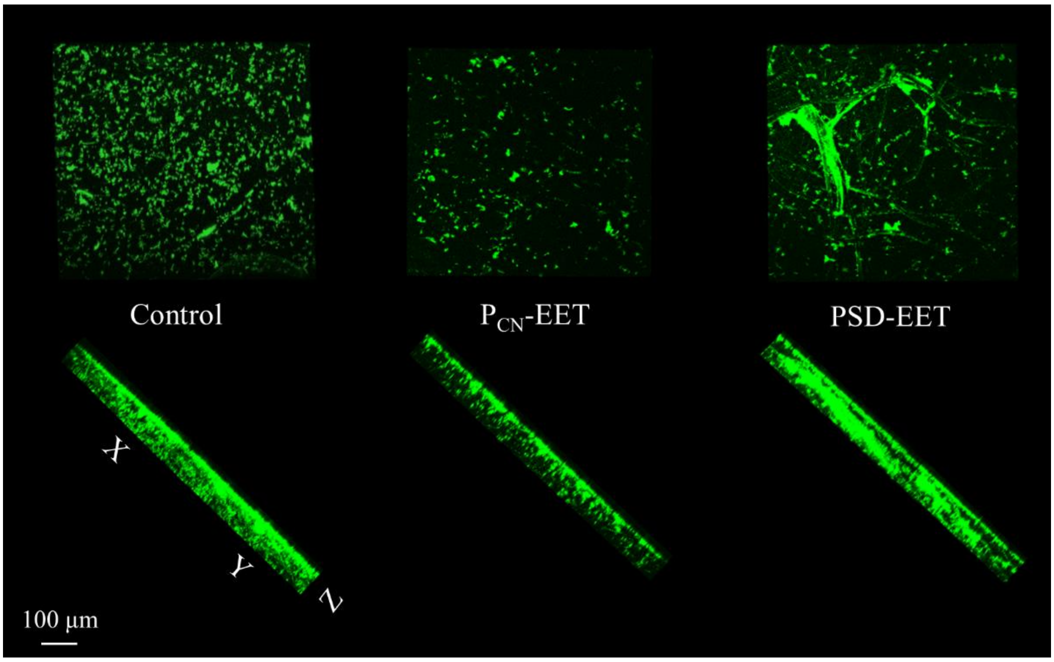

Fig. S12. Three-dimensional images of the electrode-attached biofilms formed by the three strains after staining with the green biofilm cell stain FM® 1-43. Control: WT/pYYDT.

Table S1. Strains and plasmids used in this work

Strain or plasmid	Relevant characteristics	Ref.
Strains		
Escherichia coli		
neb10β	*Δ(ara-leu) 7697 araD139 fhuA ΔlacX74 galK16 galE15 e14- ϕ80dlacZΔM15 recA1 relA1 endA1 nupG rpsL (Str*^{R}*) rph spoT1 Δ(mrr-hsdRMS-mcrBC)*	NEB
WM3064	*thrB1004 pro thi rpsL hsdS lacZΔM15 RP4-1360 Δ(araBAD)567 ΔdapA1341::*[erm pir]	(1)
Shewanella oneidensis		
MR-1	Wild-type strain	Lab stock
PSD1	MR-1 derivative, genome implanted the cassette P_{LuxI}-*luxI*-P_{LuxR}-*luxR*	This study
PSD2	MR-1 derivative, genome implanted the cassette P_{Lac}-*luxI*-*luxR*	This study
PSD3	MR-1 derivative, genome implanted the cassette P_{LuxI}-*luxI*-P_{Lac}-*luxR*	This study
PSD4	MR-1 derivative, genome implanted the cassette P_{CN}-*luxI*-P_{Lac}-*luxR*	This study
Plasmids		
pTD103	pZ vector, Kan^R, P_{LuxI}-*luxI*-P_{LuxR}-*luxR*-P_{Lux}-*sfGFP*	(2)
pYYDT	pBBR1 Broad-host plasmid, Kan^R	Lab stock
pEC01PCNOID	p15A, Kan^R, oriV, *trfA*, *lacI*-P_{CN}-Oid-GFP_{mut3b}-T_1T_2	(3)
pICE102-T7RNAP	p15A, Tet^R, *trfA*, oriV, *lacI*-P_{Lac}-*T7RNAP*	(3)
pYYDT-LuxR-GFP	pYYDT derivative, *lacI*-P_{Lac}-*luxR*-P_{Lux}-*sfGFP*-T_1T_2	This study
pYYDT-LuxI-LuxR-GFP	pYYDT derivative, P_{BAD}-*luxI*-P_{Lac}-*luxR*-P_{Lux}-*sfGFP*-T_1T_2	This study
pYYDT-P_{Lux}-GFP	pYYDT derivative, P_{Lux}-*sfGFP*-T_1T_2	This study
pYPSD1	pYYDT derivative, T_{rrnB}-P_{LuxI}-*luxI*-P_{LuxR}-*luxR*-T_1, P_{Lux}-*sfGFP*-T_1T_2	This study
pYPSD2	pYYDT derivative, T_{rrnB}-PL-*luxI*-*luxR*-T_1, P_{Lux}-*sfGFP*-T_1T_2	This study
pYPSD3	pYYDT derivative, T_{rrnB}-P_{LuxI}-*luxI*-P_{Lac}-*luxR*-T_1, P_{Lux}-*sfGFP*-T_1T_2	This study
pYPSD4	pYYDT derivative, T_{rrnB}-P_{CN}-*luxI*-P_{Lac}-*luxR*-T_1, P_{Lux}-*sfGFP*-T_1T_2	This study
pRE112	Suicide vector, Cm^R, r6k, *sacB*	(1)
pRE112-PSD1	pRE112 derivative, for implanting the cassette T_{rrnB}-P_{LuxI}-*luxI*-P_{LuxR}-*luxR*-T_1	This study
pRE112-PSD2	pRE112 derivative, for implanting the cassette T_{rrnB}-P_{Lac}-*luxI*-*luxR*-T_1	This study
pRE112-PSD3	pRE112 derivative, for implanting the cassette T_{rrnB}-P_{LuxI}-*luxI*-P_{Lac}-*luxR*-T_1	This study
pRE112-PSD4	pRE112 derivative, for implanting the cassette T_{rrnB}-P_{CN}-*luxI*-P_{Lac}-*luxR*-T_1	This study
pModuleA	pYYDT derivative, P_{Lux}-*omcA*-*mtrC*-*mtrA*-*mtrB*-T_1T_2	This study
pModuleB	pYYDT derivative, P_{Lux}-*cymA*-T_1T_2	This study
pModuleC	pYYDT derivative, P_{Lux}-*ribD*-*ribH*-*ribBA*-*ribE*-*ribC*-T_1T_2	This study
pModuleD	pYYDT derivative, P_{Lux}-*ushA*-T_1T_2	This study
pYYDT-PSD-EET	pYYDT derivative, with the modules A, B and C assembled.	This study
pYYDT-P_{CN}-EET	pYYDT derivative, substituting the P_{Lux} promoter of plasmid pYYDT-PSD-EET with P_{CN}	This study

Table S2. Primers used in this work

Primers	Sequences (5'-3')	Utilization
luxRPLUX-PYYDT F	AGCGATACAATAGTGTGACTCGACCACTCGAGGCCAGGCATC	Construct
PLAC-PYYDT R	CGTCGGAATTGCCAGCCGGGATCCGAGGTACCACAGAAATC	pYYDT-LuxR-
PYYDT-PLAC F	TGTGGTACCTCGGATCCCGGCTGGCAATTCCGACGTCATTG	GFP
luxRPLUX -PLAC R	GCGTGTTATATATCATAGCTGTTTCCTGTGTGAAATTGTTATCCG	
PLAC-luxRPLUX F	CACACAGGAAACAGCTATGATATATAACACGCAAAACTTGCGAC	
PYYDT-luxRPLUX R	TGGCCTCGAGTGGTCGAGTCACACTATTGTATCGCTGGGAATAC	
CON.PYYDT F	GATGAGCCGTGTTTTCTGGACG	
Con.Plac R	GTTCCCACTGCGATGCTGGTTG	
Con.plac F	GCCAGTTGTTGTGCCACGC	
con.Plux R	GCCGTATGAATAGGGAAACTAAACCCAGTG	
con.PLUX F	CGTTCCTGTACATAACCTTCGGGC	
CON.PYYDT R	GGAAAATGGCCGCTTTTCTGGATTC	
Blut-PYYDTLUXR F	TGAAGACGCCTAGGTCTAGGGC	Construct
Blut-PYYDTLUXR R	CCTCGAGTTAATTTTTAAAGTATGGGC	pYYDT-LuxI-L
PYYDTLUXR-pbadlu xI F	GCCCATACTTTAAAAATTAACTCGAGGTATAAACGCAGAAAGGCCCACCCG	uxR-GFP
PYYDTLUXR-pbadlu xI R	GCCCTAGACCTAGGCGTCTTCATCATGAATTAGTCTCGGACATTCTG	
seq.luxI F	GAGCACCACCCGGATGCCTATCG	
seq.luxR R	CTCACACAATGTATACATCACGGCAGAC	
seq.luxR F	CATCAGGTCTTATCACTGGGTTTAG	
SEQ.LUX I R	GCTCAAAGATAAATAACTCTGCTAGTG	
BLT-PYT F	TTGCTTTCGAACCCCAGAGTCCCGCTCAGAAGAACTCGTCAAGAAGGCGATA	Construct
BLT-PYT R	TCTTCACGGGATCCGAGGTACCACAGAAATCATCCTTAG	pYYDT-P_{Lux}-G
BLT-PLUX F	ACCTCGGATCCCGTGAAGACGCCTAGGTCTAGGGCGGCGGAT	FP
BLT-PLUX R	GGACTCTGGGGTTCGAAAGCAAATTCGACCCGGTCGTCG	
S.PYRIPL F	TCAGGCATTTGAGAAGCACACGGTC	
S.sfGFP R	CACACAATGTATACATCACGGCAGAC	
S.sfGFP F	CTTCAACGTTGTGGCGAATTTTGAAG	
S.PYRIPL R	GCGGACCGCTATCAGGACAT	
BLT-PYTRI F	CAGCCGGGATCCGAGGTACCACAGAAATC	Construct
BLT-PYTRI R	CGGCGGATTTGTCCTACTCAGGAGAGC	pYPSD1
PYTRI-oriQS F	TCTCCTGAGTAGGACAAATCCGCCGCTCGAGTTAATTTTTAAAGTATGGGCAATC	
PYTRI-oriQS 2R	CTGTGGTACCTCGGATCCCGGCTGGTGAGCTGATACCGCTCGCCGC	
S.PYRIPL 2F	CAAAGATGACGGGACCTACAAG	
S.LuR R	ACGGCTAACAATGGCTTCGGAATGC	
S.LuI F	GCTCAAAGATAAATAACTCTGCTAGTG	
S.PYTRI 2R	TCAGGCATTTGAGAAGCACACGGTC	
PYRI-PL F	TTCTGTGGTACCTCGGATCCCGGCTGCGGGACTCTGGGGTTCGAAATG	Construct
LuR-PL R	TAATGGATTGACATTTGTTCCACACAACATACGAGCCGGAAG	pYPSD2
PL-LuR F	GTATGTTGTGTGGAACAAATGTCAATCCATTACCGTTTTAATGATA	

LuI-LuR R	CTCCTTAGAGCTCGAATTTAATTTTTAAAGTATGGGCAATCAATTGC	
LuR-LuI F	CATACTTTAAAAATTAAATTCGAGCTCTAAGGAGGTTATAAAAAATG	
PYTRI-luI R	TCTCCTGAGTAGGACAAATCCGCCGCGCATGCTCCTCTAGACTCGATTAAAGGGC	
S.PL R	TTCCACACAACATACGAGCCGGAAG	
S.LuR F	CTAAACCCAGTGATAAGACCTGATG	
S.LuR R	ACGGCTAACAATGGCTTCGGAATGC	
S.LuI R	TGGGAGCACTCTGTTGACCAAGC	
S.LuI F	GCTCAAAGATAAATAACTCTGCTAGTG	
PLu-PL R	CCTGTACGATCCTACAGGTGCTGCGGGACTCTGGGGTTCGAAATG	Construct
PL-PLu F	CGAACCCCAGAGTCCCGCAGCACCTGTAGGATCGTACAGGTTTACG	pYPSD3
PCN-PL 22R	ACATATCCTCTGGTGCAGCGGGACTCTGGGGTTCGAAATG	Construct
PL-PCN 22F	CGAACCCCAGAGTCCCGCTGCACCAGAGGATATGTCAAGCATATAGGAC	pYPSD4
LuI-PCN R	CTCCTCTTTAATGAATTCGGGGTGAAAAAGCACCTGTATACGCCCCT	
PCN-LuI F	ATACAGGTGCTTTTTCACCCCGAATTCATTAAAGAGGAGAAAGGTACCATG	
S.Pcn R	CTGTATACGCCCCTTCTTATAGGACG	
112-PSD1 F	CAATTCCGACGTCATTGCGTTGCGCGTGAGCTGATACCGCTCGCCGCA	Construct
112-PSD1 R	GGCCTTTCTGCGTTTATACCTCGAGCTCGAGTTAATTTTTAAAGTATGGGCAATC	pRE112-PSD1
Blut-PLAT(SITE2) F	CTCGAGGTATAAACGCAGAAAGGCCCA	
Blut-PLAT(SITE2) R	GCGCAACGCAATGACGTCGGAATTG	
seq.LA(site2) F	GCGATTCAGGCTGATGAAGGTGC	
S.LuI F	GCTCAAAGATAAATAACTCTGCTAGTG	
S.LuR R	ACGGCTAACAATGGCTTCGGAATGC	
S. LA(site2) R	GAGGTACAGCAGGCAATCAGAGTCAAC	
112-PSD2 F	CAATTCCGACGTCATTGCGTTGCGCGCGGGACTCTGGGGTTCGAAATGACCG	Construct
112-PSD2 R	GCCTTTCTGCGTTTATACCTCGAGCTAGTACTCGAGTCTAGATTAAAGGGCG	pRE112-PSD2
S.PL R	TTCCACACAACATACGAGCCGGAAG	
S.LuI F	GCTCAAAGATAAATAACTCTGCTAGTG	
112-PSD3 F	CAATTCCGACGTCATTGCGTTGCGCAGTGAGCTGATACCGCTCGCCGC	Construct
112-PSD3 R	GGCCTTTCTGCGTTTATACCTCGAGCTCGAGTTAATTTTTAAAGTATGGGCAATC	pRE112-PSD3
S.LuI F	GCTCAAAGATAAATAACTCTGCTAGTG	
S.LuR R	ACGGCTAACAATGGCTTCGGAATGC	
112-PSD4 F	AATTCCGACGTCATTGCGTTGCGCAGTGAGCTGATACCGCTCGCCGC	Construct
112-PSD4 R	GCCTTTCTGCGTTTATACCTCGAGCTCGAGTTAATTTTTAAAGTATGGGCAATC	pRE112-PSD4
S.LuI F	GCTCAAAGATAAATAACTCTGCTAGTG	
S.LuR R	ACGGCTAACAATGGCTTCGGAATGC	
BLT-PYYT F	CTCGACCACTCGAGGCCAGGCATCAAATAAAACG	Construct
BLT-PYYT R	GAGGTACCACAGAAATCATCCTTAGCG	pModuleA
PYYT-TTs F	CTAAGGATGATTTCTGTGGTACCTCCTAAACTGACAGGCATCAAATTAAGC	
BLT-TTs R	CACCACCGAAATTCAGTAAGCAG	
TTs-omcA F	CTTTCTGCTTACTGAATTTCGGTGGTGATGATGAAACGGTTCAATTTCAATACCGC	
Mtr-omcA R	AGGTTTGGCATTGCTTTTAGTTACCGTGTGCTTCCATCAATTG	
OmcA-mtr F	AGCACACGGTAACTAAAAGCAATGCCAAACCTATGCAGGGAAAAAAATG	
PYT-mtr R	ATTTGATGCCTGGCCTCGAGTGGTCGAGGATTAGAGTTTGTAACTCATGCTCAGCATC	
BLT-omcmtr F	ATGATGAAACGGTTCAATTTCAATACCGCAAC	

BLT-PYTBB R	CACCACCGAAATTCAGTAAGCAGAAAGTC	
PYTBB-PLux F	GACTTTCTGCTTACTGAATTTCGGTGGTGCCTCGAGTGGTCGAGTCACACTATTG	
Omcmtr-PLux R	CGGTATTGAAATTGAACCGTTTCATCATGGTACCTTTCTCCTCTTTAATGAATTCATTC	
S.PY F	CTATCGGCTGTAGCCGCCTCG	
S.TTs R	CACCACCGAAATTCAGTAAGCAG	
S.omcA R	CTCCCACTTTTTCTTTAACGGGAG	
S.omcA F	GATTGTGCAACATGTCATACTGCTG	
S.mtr R	GCGCTATTACCTGGGCCTGTCG	
S.mtr F	TGGAGAACAAGTTACGCCTAGGAC	
SEQ.PYYT R	TGGATTCATCGACTGTGGCCGGC	
S.PLux F	CCTCGAGTGGTCGAGTCACACTATTG	
S.omcA R	CTCCCACTTTTTCTTTAACGGGAG	
S.PY F	CTATCGGCTGTAGCCGCCTCG	
S.PLux R	GGTACCTTTCTCCTCTTTAATGAATTCATTC	
TTs-cymA F	CTTTCTGCTTACTGAATTTCGGTGGTGATGAACTGGCGTGCACTATTTAAACCCAG	Construct
PYT-cymA R	TGATGCCTGGCCTCGAGTGGTCGAGTTATCCTTTTGGATAGGGGTGAGCGAC	pModuleB
BLT-cymA F	ATGAACTGGCGTGCACTATTTAAACCCAG	
cymA-Plux R	GGGTTTAAATAGTGCACGCCAGTTCATGGTACCTTTCTCCTCTTTAATGAATTCATTC	
S.cymA R	ACCAAGCTTGAGTGTTAAAGCCATC	
S.cymA F	TGCGTTCTGTATGTCTTGCCATAGC	
S.cymA R	ACCAAGCTTGAGTGTTAAAGCCATC	
S.PY F	CTATCGGCTGTAGCCGCCTCG	
TTs-ribD F	CTTTCTGCTTACTGAATTTCGGTGGTGATGAATTGGTCAGTACTCGATAACCAAATG	Construct
ribE-ribBA R	TAGCTAAAGCACCCAGTTACTCAGCTACGCATTCGGTCAC	pModuleC
ribBA-ribE F	ATGCGTAGCTGAGTAACTGGGTGCTTTAGCTAAATTAGGTAAG	
ribC-ribE R	ATTCGTTAAAGGCTGTATTACAACTGTTGTTCAAGCTGTTGCAG	
ribE-ribC F	TTGAACAACAGTTGTAATACAGCCTTTAACGAATAAGCGAGC	
PYT-ribC R	GATGCCTGGCCTCGAGTGGTCGAGTTAAGATAGTCCGTTAAAACGTTTAGCCAATA	
BLT-Flavin F	ATGAATTGGTCAGTACTCGATAACCAAATG	
Flavin-PLux R	ATTTGGTTATCGAGTACTGACCAATTCATGGTACCTTTCTCCTCTTTAATGAATTCATTC	
S.ribD R	CACGATGCAAGCCCACATCCAC	
S.ribBA F	CGATATGGTGACCTTTAGGGATAC	
S.ribE R	GACCTTTGTTACATTCGCCTGC	
S.ribE F	GGTGCACTTGACACGCTGAAACG	
S.ribC R	TGCCAAAAGTGAGCGAGCGTTCA	
S.ribC F	GATTGGTGGTCATATCTTATCAGGTC	
TTs-ushA F	CTGCTTACTGAATTTCGGTGGTGATGACAAATATGCTTATTAAAGGACTCATTG	Construct
PYT-ushA R	TATTTGATGCCTGGCCTCGAGTGGTCGAGTTACTTCGCTGTAATCTTACAACCTTCTAC	pModuleD
BLT-ushA F	ATGACAAATATGCTTATTAAAGGACTCATTGC	
ushA-PLux R	ATGAGTCCTTTAATAAGCATATTTGTCATGGTACCTTTCTCCTCTTTAATGAATTCATTC	
S.ushA R	ACCAGTAAAGTCAGGAATCGCATC	
S.ushA f	GATTATTTAGGTGCAGTGGGTAG	
SEQ.PYYT R	TGGATTCATCGACTGTGGCCGGC	
S.ushA R	ACCAGTAAAGTCAGGAATCGCATC	

S.PY F	CTATCGGCTGTAGCCGCCTCG	
PL-QS-CymA.F	ATCTTAACTCGACCACTCGAGGCCAGGCA	Construct
PL-QS-CymA.R	CGTTTCATCATGAGTTATCCTTTTGGATAGGGGTGAGCGACACC	pYYDT-PSD-E
PL-QS-Mtr.F	AGGATAACTCATGATGAAACGGTTCAATTTCAATACCGCAACAAAAGC	ET
PL-QS-Mtr.R	TGACCAATTCATGATTAGAGTTTGTAACTCATGCTCAGCATCAGCATTTGTGC	
PL-QS-Rib.F	AAACTCTAATCATGAATTGGTCAGTACTCGATAACCAAATGATGAGCCG	
PL-QS-Rib.R	TCGAGTGGTCGAGTTAAGATAGTCCGTTAAAACGTTTAGC	
QS-PCN.F	AGAAAGGTACCATGAACTGGCGTGCACTATTTAAACCCAGC	Construct
QS-PCN.R	CTCTGGTGCACACCACCGAAATTCAGTAAGCAGAAAGTCAAAAGC	pYYDT-P_{CN}-E
PCN-QS.F	TTCGGTGGTGTGCACCAGAGGATATGTCAAGCATATAGGACGTATACGGG	ET
PCN-QS.R	GTGCACGCCAGTTCATGGTACCTTTCTCCTCTTTAATGAATTCGG	
RT-gyrB-F	F- CAGTGGAACGACGGCTACCAAG	qRT-PCR
RT-gyrB-R	R- CGGGCATCATCGCCAGTCG	primers
RT-cymA-F	F- ACACTGAACAGTAACCCCGGC	
RT-cymA-R	R- GCGCTACTGGTTGTTGGTATCG	
RT-omcA-F	F- ACCCAAATTACGGCACCATTG	
RT-omcA-R	R- AGGCGTACAATCTGGCACTG	
RT-mtrC-F	F- GTACTGGCTGCCAACTGTGTG	
RT-mtrC-R	R- CTACGGCCTCATTGGTGGC	
RT-mtrA-F	F- CTCAGTGACGGGTGCATGC	
RT-mtrA-R	R- GTCACCCACTCAAATGGGCAC	
RT-mtrB-F	F- CGGCGATATCATTTGCGGCG	
RT-mtrB-R	R- GTCAGACAAGGTATCACTGGCGAC	
RT-ribD-F	F- GTAGATTACGTCCTTGGCCG	
RT-ribD-R	R- TACCAGCCATTGAGGGTCG	
RT-ribE-F	F- GACGTGCAGCCAGCGGTAAC	
RT-ribE-R	R- GCGAAGAATGCCAAAGTTGCG	
RT-ribH-F	F- GGCTCACGCCATCAATCGTG	
RT-ribH-R	R- CGGCCATATGGTGAGTGGTC	
RT-ribBA-F	F- GGCGAGGATTTCACTGGAATG	
RT-ribBA-R	R- GCCTGACTGTTTAGTACGGGTAC	
RT-ribC-F	F- GATTTGCATGAGGGACTTGCG	
RT-ribC-R	R- CCACGGATAAACCCGCCAG	

Strain and cultivation conditions

The mineral medium was prepared according to the method described by Marsili *et al.* (4). It contained 20 mM lactate, 0.5 g/L casamino acid, 10 mM HEPES ($C_8H_{18}N_2O_4S$), 0.46 g/L NaCl, 0.225 g/L K_2HPO_4, 0.225 g/L KH_2PO_4, 0.225 g/L $(NH_4)_2SO_4$, and 0.117 g/L $MgSO_4{\cdot}7H_2O$. Trace elements were supplemented with 10 mL stock solution, which contained 1.5 g/L nitrilotriacetic acid (NTA), 0.1 g/L $MnCl_2{\cdot}7H_2O$, 0.3 g/L $FeSO_4{\cdot}7H_2O$, 0.17 g/L $CoCl_2{\cdot}6H_2O$, 0.1 g/L $ZnCl_2$, 0.04 g/L $CuSO_4{\cdot}5H_2O$, 0.005 g/L $AlK(SO_4)_2{\cdot}12H_2O$, 0.005 g/L H_3BO_3, 0.09 g/L Na_2MoO_4, 0.12 g/L $NiCl_2$, 0.02 g/L $NaWO_4{\cdot}4H_2O$, and 0.1 g/L Na_2SeO_3.

Genetic manipulation, plasmid construction and conjugation, and host construction

The genomic DNA was isolated using E.Z.N.A. Bacterial DNA Isolation Kit (OMEGA, Bio-Tek, USA). RNA was extracted with the RNAiso Plus Kit (Takara Co., China), and the plasmid extraction was conducted with Plasmid Mini Kit (OMEGA, Bio-Tek, USA), following the manufacturers' instructions. The DNA or RNA concentrations were determined by Nanodrop (Thermo Fisher Scientific, USA). DNA restriction enzymes, Q5 High-Fidelity DNA polymerase, Taq DNA ligase, and T5 Exonuclease were purchased from New England BioLabs (NEB, USA). The PCR amplifications for plasmid constructions were conducted with Q5 High-Fidelity DNA polymerase. PCR verification was performed with MonAmp 2×Taq Mix (Monad Biotech Co., China). Sanger DNA sequencing was submitted to commercial companies performing on 3730xl DNA Analyzers (General Biosystems Co., China).

The gene expression levels were determined with quantitative reverse transcription-polymerase chain reaction (qRT-PCR) using the housekeeping gene *gyrB* as the internal reference. The PrimeScript II first Strand cDNA Synthesis Kit (Takara Co., China) was used for RNA inverse transcription, and the SYBR Premix Ex Taq (Takara Co., China) was used for qRT-PCR determination with the StepOne real-time PCR system (Applied Biosystems Inc., USA).

E. coli electro-competent cells were prepared with the method described earlier (5). Briefly, the overnight *E. coli* culture was inoculated into fresh 2×YT media at 1% (v/v). When the cell density of OD_{600} reached ~ 0.5-0.6, it was immediately chilled on ice. *E. coli* cells were then centrifugated at 4000 rpm and washed twice with ice-cold 10% glycerol. Lastly, cells were concentrated by 100-fold and splitted into 50 μL/tube for utilization.

Electroporation was completed with a pre-chilled 2-mm gap electroporation cuvette (Bio-Rad, USA) and electroporated at 2.5 kV with a MicroPulser (Bio-Rad, USA). 1 mL of 2-YT medium was added immediately to the shocked cells and recovered for 45-60 mins before plating on 2×YT agar with appropriate antibiotics.

Conjugation was used to introduce constructed plasmids into *S. oneidensis* cells. The plasmids were initially transformed into the donor strain *E. coli* WM3064. The same volumes of 1-1.5 mL of donor cells and *S. oneidensis* (recipient cells) were mixed evenly and centrifugated at 4000 rpm for collection. Then, they were resuspended in 1 mL 2×YT medium and plated on 2×YT agar (with DAP) for conjugation (lasting 24-48 h). The microbial layers were washed off from the agar

plate with liquid 2×YT without DAP. Lastly, the cell suspension was diluted and spread on 2×YT agar plates with appropriate antibiotics.

To construct the PSD system, the cassette P_{Lux}-*sfGFP* were amplified from plasmid pTD103 (2), and cloned into plasmid pYYDT to generate the plasmid pYYDT-P_{Lux}-GFP. The cassette *lacI*-P_{Lac} was amplified from plasmid pICE102-T7RNAP (3), and *luxR* was amplified from pTD103; they were both cloned into pYYDT-P_{Lux}-GFP to generate plasmid pYYDT-LuxR-GFP. The promoter P_{BAD} was amplified from plasmid pEC101, and the *luxI* was amplified from plasmid pTD103; they were assembled into pYYDT-LuxR-GFP to generate plasmid pYYDT-LuxI-LuxR-GFP.

To obtain the autonomously regulated PSD system, the cassette T_{rrnB}-P_{LuxI}-*luxI*-P_{LuxR}-*luxR*-T_1 was amplified from plasmid pTD103, and inserted into plasmid pYYDT-P_{Lux}-GFP to generate plasmid pYPSD1. The promoter $P_{Lac,}$ *luxI*, and *luxR* were assembled in tandem into pYYDT-P_{Lux}-GFP to generate plasmid pYPSD2. The P_{Lac} promoter was used to substitute the original promoter of *luxR* in pYPSD1 to generate plasmid pYPSD3. The original promoters of *luxI* and *luxR* from pPSD1 were substituted by the constitutive promoter P_{CN} (amplified from plasmid pEC01PCNOID) and P_{Lac}, respectively, to generate plasmid pYPSD4.

To construct the decision-making unit implanted strains, the *luxI*-*luxR* cassette variants were amplified from pYPSD1, pYPSD2, pYPSD3, and pYPSD4, respectively; the homologous arms flanking the targeted integration locus were amplified; they were inserted into plasmid pRE112 correspondingly, to generate plasmid pRE112-PSD1,

pRE112-PSD2, pRE112-PSD3, and pRE112-PSD4, respectively. Next, they were transferred into *S. oneidensis* via. conjugation, and spread on Cm^R plate for selection of the first-crossover events. The positive colonies were then picked and transferred for the selection of the second crossover events on sucrose plates. The formed colonies were then screened and checked with PCR verification.

The four decision implementation modules were constructed based on plasmid pYYDT-P_{Lux}-GFP. The genes *omcA, mtrC, mtrA, and mtrB* were assembled and placed under the control of the P_{Lux} promoter to generate plasmid pModuleA. The *cymA* gene was placed under the P_{Lux} control to generate plasmid pModuleB. The genes *ribD, ribH, ribBA, ribE*, and *ribC* were assembled and placed under the P_{Lux} control to generate plasmid pModuleC. The *ushA* gene was placed under the P_{Lux} control to generate plasmid pModuleD. Then, modules A, B, and C were assembled to generate plasmid pYYDT-PSD-EET. The promoter P_{Lux} of plasmid pYYDT-PSD-EET was substituted with promoter P_{CN} to generate plasmid pYYDT-P_{CN}-EET.

References

1. R. A. Edwards, L. H. Keller, D. M. Schifferli, Improved allelic exchange vectors and their use to analyze 987P fimbria gene expression. *Gene* **207**, 149-157 (1998).
2. A. Prindle *et al.*, A sensing array of radically coupled genetic 'biopixels'. *Nature* **481**, 39-44 (2012).
3. Q. Tang, T. Lu, S.-J. Liu, Developing a synthetic biology toolkit for *Comamonas testosteroni*, an emerging cellular chassis for bioremediation. *ACS Synth. Biol.* **7**, 1753-1762 (2018).
4. E. Marsili *et al.*, *Shewanella* secretes flavins that mediate extracellular electron transfer. *Proc. Natl. Acad. Sci. U.S.A.* **105**, 3968-3973 (2008).
5. J. Li et al., Rediverting electron flux with an engineered CRISPR-ddAsCpf1 system to enhance the pollutant degradation capacity of *Shewanella oneidensis.* *Environ. Sci. Technol.* **54**, 3599-3608 (2020).
